# Fic Proteins of *Campylobacter fetus* subsp. *venerealis* Form a Network of Functional Toxin–Antitoxin Systems

**DOI:** 10.3389/fmicb.2017.01965

**Published:** 2017-10-17

**Authors:** Hanna Sprenger, Sabine Kienesberger, Brigitte Pertschy, Lisa Pöltl, Bettina Konrad, Priya Bhutada, Dina Vorkapic, Denise Atzmüller, Florian Feist, Christoph Högenauer, Gregor Gorkiewicz, Ellen L. Zechner

**Affiliations:** ^1^Institute of Molecular Biosciences, University of Graz, Graz, Austria; ^2^Institute of Pathology, Medical University of Graz, Graz, Austria; ^3^Division of Gastroenterology and Hepatology, Medical University of Graz, Graz, Austria; ^4^BioTechMed-Graz, Graz, Austria; ^5^Vehicle Safety Institute, Graz University of Technology, Graz, Austria

**Keywords:** post-translational modification, adenylylation, toxin–antitoxin module, bacterial effector protein, bacterial evolution, niche adaptation, urogenital tract, virulence

## Abstract

Enzymes containing the FIC (filamentation induced by cyclic AMP) domain catalyze post-translational modifications of target proteins. In bacteria the activity of some Fic proteins resembles classical toxin–antitoxin (TA) systems. An excess of toxin over neutralizing antitoxin can enable bacteria to survive some stress conditions by slowing metabolic processes and promoting dormancy. The cell can return to normal growth when sufficient antitoxin is present to block toxin activity. *Fic* genes of the human and animal pathogen *Campylobacter fetus* are significantly associated with just one subspecies, which is specifically adapted to the urogenital tract. Here, we demonstrate that the *fic* genes of virulent isolate *C. fetus* subsp. *venerealis* 84-112 form multiple TA systems. Expression of the toxins in *Escherichia coli* caused filamentation and growth inhibition phenotypes reversible by concomitant antitoxin expression. Key active site residues involved in adenylylation by Fic proteins are conserved in Fic1, Fic3 and Fic4, but degenerated in Fic2. We show that both Fic3 and the non-canonical Fic2 disrupt assembly and function of *E. coli* ribosomes when expressed independently of a trans-acting antitoxin. Toxicity of the Fic proteins is controlled by different mechanisms. The first involves intramolecular regulation by an inhibitory helix typical for Fic proteins. The second is an unusual neutralization by heterologous Fic–Fic protein interactions. Moreover, a small interacting antitoxin called Fic inhibitory protein 3, which appears unrelated to known Fic antitoxins, has the novel capacity to bind and neutralize Fic toxins encoded in *cis* and at distant sites. These findings reveal a remarkable system of functional crosstalk occurring between Fic proteins expressed from chromosomal and extrachromosomal modules. Conservation of *fic* genes in other bacteria that either inhabit or establish pathology in the urogenital tract of humans and animals underscores the significance of these factors for niche-specific adaptation and virulence.

## Introduction

The genus *Campylobacter* comprises ecologically diverse species that colonize humans and animals. *Campylobacter jejuni* is known as the leading cause of human bacterial diarrhea worldwide. Other *Campylobacter* species, including *Campylobacter fetus*, are increasingly recognized as important human and animal pathogens (Lastovica and Allos, 2008; Man, 2011; Bullman et al., 2013). *C. fetus* is intriguing because although the two subspecies associated with mammals, *C. fetus* subsp. *fetus* and *C. fetus* subsp. *venerealis*, are highly related at the genome level, they exhibit quite different niche adaptations. *C. fetus* subsp. *fetus* has a broad host range (Skirrow and Benjamin, 1980; Harvey and Greenwood, 1985; Logue et al., 2003). In humans it causes gastrointestinal disease and belongs to the *Campylobacter* spp. most frequently associated with bacteremia (Lastovica and Allos, 2008; Man, 2011). By contrast, *C. fetus* subsp. *venerealis* is a host-restricted veterinary pathogen adapted to the urogenital tract of cattle (Blaser et al., 2008). Current understanding of the pathogenesis of emerging *Campylobacter* spp. is quite limited.

Recent comparative genomics of *C. fetus* subspecies revealed genetic determinants potentially contributing to this species’ niche preferences and pathogenicity (Kienesberger et al., 2014; Graaf-van Bloois et al., 2016). Strikingly, *C. fetus* genomes encode multiple bacterial type IV secretion systems (T4SS), which generally contribute to pathogenicity by transferring specific protein and DNA substrates to recipient cells (Christie et al., 2014). One *C. fetus* T4SS has been evaluated experimentally and linked to virulence (Gorkiewicz et al., 2010). The conserved T4SS-encoding regions of *C. fetus* genomes fit into three phylogenetically different groups: one located exclusively on the chromosome, one observed exclusively on plasmids and a third located on both (Graaf-van Bloois et al., 2016). These authors further showed that both genes encoding T4SS components and genes encoding FIC domain proteins are significantly associated with the *C. fetus* subsp. *venerealis*. In the current study we focus on the function of the *fic* genes.

The Fido domain superfamily is composed of members of the FIC (filamentation induced by cAMP) and the Doc (death on curing) protein families and is common in all domains of life (Kinch et al., 2009). Proteins of the combined family contain a conserved motif [HPFx(D/E)GN(G/K)R]. Work in recent years has revealed that enzymes of the family catalyze post-translational modifications of proteins by addition of AMP, other nucleoside monophosphates, phosphocholine, or phosphate to a functionally critical amino acid (as reviewed in Cruz and Woychik, 2014; Garcia-Pino et al., 2014; Roy and Cherfils, 2015; Harms et al., 2016b). Since activities of the target proteins are typically altered as a result, Fido proteins are recognized as important regulators of metabolic functions.

Phylogenetic analysis of the superfamily places the paradigm Doc toxin of bacteriophage P1 in subfamily I (Garcia-Pino et al., 2014). Doc toxin is structurally similar to Fic proteins (Garcia-Pino et al., 2008); however, variation in the catalytic motif (K in place of the second G) confers kinase activity in place of NMP transfer activity (Castro-Roa et al., 2013; Cruz et al., 2014). Phosphorylation of translation elongation factor Tu by Doc leads to rapid translation arrest in *Escherichia coli* (Garcia-Pino et al., 2008; Liu et al., 2008; Castro-Roa et al., 2013).

Interest in the FIC protein subfamily has been fueled by the observation that bacterial pathogens secrete Fic enzymes to modify host proteins (Pan et al., 2008; Worby et al., 2009; Yarbrough et al., 2009; Mukherjee et al., 2011). Cell to cell transfer can be direct via type III or type IV secretion (Roy and Cherfils, 2015). In the host, Fic effector proteins contribute to bacterial pathogenicity by modifying proteins important to signaling (Roy and Mukherjee, 2009; Woolery et al., 2010). Fic effectors contain the canonical catalytic motif [HxFx(D/E)GNGRxxR] and initial studies showed that a typical reaction inactivates host GTPases by nucleotidyl transfer to a hydroxyl group of the protein side chain (Garcia-Pino et al., 2014; Roy and Cherfils, 2015). Several secreted FIC proteins transfer AMP in a reaction called adenylylation (Worby et al., 2009; Yarbrough et al., 2009; Zekarias et al., 2010; Palanivelu et al., 2011), but variation within the canonical core motif can alter enzyme activity (Mukherjee et al., 2011; Engel et al., 2012).

The targets of FIC enzyme modification are not restricted to proteins expressed by the host. However, their functions and regulation in producing bacteria are still poorly understood. *E. coli* has been used as a surrogate producer to gain insights into the activities of FIC proteins in bacteria. One function that has emerged from these studies is that Fic proteins act as toxin–antitoxin (TA) modules (Harms et al., 2016b). Bacterial TA systems play a major role in cellular adaptation to stress and persistence (Hayes and Van Melderen, 2011; Goeders and Van Melderen, 2014; Harms et al., 2016a). Activation of the toxin can cause slow cell growth or arrest the cell cycle allowing bacteria to enter a dormant state. Mechanistic understanding of TA activity has been developed with prototypic modules such as *phd-doc* of bacteriophage P1 (Lehnherr et al., 1993; Castro-Roa et al., 2013). Generally the toxin component is directed against the producing cell and interferes with bacterial physiology. Cellular processes inhibited by type II TA toxins include protein synthesis, cell wall synthesis, assembly of cytoskeletal structures and DNA topoisomerase action (Hayes and Van Melderen, 2011; Yamaguchi and Inouye, 2011; Goeders and Van Melderen, 2014; Harms et al., 2016b). The antitoxin component reversibly inactivates the toxin and/or regulates its expression. Unlike the toxin, the antitoxin is biochemically unstable so that, unless the antitoxin is continuously expressed, the free toxin forces the bacterial cell into a reversible dormant state or even kills the cell (Leplae et al., 2011; Goeders and Van Melderen, 2014). The TA system of bacteriophage P1 helps to maintain the lysogen through post-segregational killing of cells that are cured of the prophage (Lehnherr et al., 1993). Homologous *phd*-*doc* modules are also present on bacterial chromosomes and evidence thus far suggests a role for these systems in the formation of persister cells under stress conditions (Maisonneuve and Gerdes, 2014). Moreover, evidence is emerging that TA modules help bacteria overcome stress imposed by host colonization, early stages of infection and survival within host cells (Norton and Mulvey, 2012; Ren et al., 2012, 2014; De la Cruz et al., 2013; Lobato-Marquez et al., 2015). They can stabilize mobile genetic elements encoding virulence factors and contribute directly to virulence (see Lobato-Marquez et al., 2016 for a comprehensive review).

*Campylobacter* genomes generally lack homologs of prototypical TA systems (Shao et al., 2011). To date, only two TA systems (both located on a plasmid in *C. jejuni*) have been described (Shen et al., 2016). Given the general importance of TA systems in bacteria we asked whether the multiple *fic* genes in *C. fetus* fulfill this important role. Here, we show that the Fic proteins of *C. fetus* subsp. *venerealis* 84-112 indeed form TA systems with the capacity to disrupt the bacterial translational machinery. We further show that *fic* modules located on the chromosome and extrachromosomal DNA functionally interact. *Fic* homologs are genetically conserved in *C. fetus* subsp. *venerealis* isolates and in other human and animal urogenital pathogens, underscoring the significance of these factors for niche-specific adaptation.

## Materials and Methods

### Bacteria

Strains used in this study are listed in Supplementary Table S1. *E. coli* and *Campylobacter* strains were grown as previously described (Kienesberger et al., 2007). Antibiotics were added to final concentrations of 100 μg ml^-1^ ampicillin, 75 μg ml^-1^ nalidixic acid, and either 12.5 or 25 μg ml^-1^ chloramphenicol, 40 or 25 μg ml^-1^ kanamycin for *E. coli* or *Campylobacter* cultivation, respectively.

### Construction of Plasmids

Plasmids and oligonucleotides used in this study are listed in Supplementary Tables S1, S2. For expression in *E. coli*, genes of interest were amplified with PCR and the fragments were ligated to pBAD24 vector derivatives with distinct antibiotic resistance genes (see Supplementary Tables S1, S2).

### Structure Predictions

For 3D structure prediction amino acid sequences (CDF65254.1, CDF65253.1, CDF65920.1, and CDF65967.1) were analyzed using the Phyre2 web portal (Rollins and Colwell, 1986). The output files were rendered with PyMOL (Schrodinger, 2010). Templates for Fic protein fold recognition via Phyre2 are listed in Supplementary Table S3.

### *E. coli* Growth/Rescue Assays

*Escherichia coli* DH5α harboring pBAD plasmids with *fic* genes were grown with shaking overnight at 37°C in LB-broth supplemented with appropriate antibiotics and 0.2% glucose to repress expression, or 0.05% arabinose to induce expression via the P_BAD_ promoter. Bacterial growth was either monitored by survival plating of bacteria grown in 100 ml LB-broth or in 24-well plates with a culture volume of 1 ml per well and starting optical density measured at 600 nm (OD_600_) of 0.05. Plates were incubated at 37°C under shaking at 180 rpm. OD_600_ was measured hourly in triplicate. Determination of colony forming units (CFUs) normally corresponded with OD_600_ measurements except at late time points where the CFU count of filamentous cells remained low.

### Microscopy

Cultures of *E. coli* DH5alpha harboring pBAD derivatives with a starting OD_600_ of 0.05 were grown in LB-broth with 0.05% arabinose for 2 h. Cells were harvested, suspended in 1x phosphate buffered saline (PBS), pH 7.4 and incubated with Nile red for up to 60 min at room temperature in the dark. For immediate microscopy, 1 μl of the pellet was applied to an agar slide (1% agar solution poured on microscopy slide) to immobilize the cells. For later imaging, cells were fixed with 0.4% formaldehyde, before collected by centrifugation, resuspended in 1x PBS, pH 7.4 and stained as described before. Confocal microscopy was performed on a LEICA AOBS SP2 MP microscope (380 nm extinction, 510 nm emission).

### Co-immunoprecipitation and Western Analysis

Pairs of FLAG-tagged and hemagglutinin (HA)-tagged proteins were co-expressed in *E. coli* C41(DE3) from respective plasmids (Supplementary Table S1). 100 ml LB broth supplemented with 0.2% glucose was inoculated with overnight cultures to OD_600_ of 0.1. When cultures reached OD_600_ = 0.5–0.8 protein expression was induced with 0.05% arabinose for 2 h. Thirty OD of cells were pelleted and washed with 50 ml buffer A (50 mM Tris-HCl pH 6.8, 100 mM NaCl). Cell lysis was performed as previously described (Gruber et al., 2016) except that the formaldehyde crosslinking step was omitted for this study. All further steps were as in Gruber et al. (2016). For protein detection, OD_600_ 0.015–0.05 equivalents of lysate and pull-down fractions were mixed with sample buffer containing DTT (0.09%) and SDS (0.1%), heated at 95°C for 10 min and resolved on SDS-PAGE (12.5%, Hoefer) or NUPAGE (12%, MES buffer, Invitrogen) gels. Proteins were transferred for 1.5 h onto PVDF membranes. Blocking was done overnight at 4°C in TST: (0.5 M Tris-HCl pH 7.5, 1.5 M NaCl, 1% Tween-20) supplemented with 3% milk powder. FLAG-tagged proteins were detected with HRP-conjugated α-FLAG antibody (A8592, Sigma) and HA-tagged proteins with HRP-conjugated α-HA antibody (12013819001, Roche). After washing (3 min × 10 min) with 1x TST blots were developed with ECL (Bio-Rad) according to the manufacturer’s instructions (1:5 dilution in ddH_2_O of substrate for FLAG detection).

### Ribosome Profiles

*Escherichia coli* DH5α with plasmids were grown in 100 ml LB-broth to an OD_600_ of 0.4 to 0.6, then shifted to medium containing 0.05% arabinose for 1 h to induce expression. 30 s prior to cell harvest, chloramphenicol was added to a final concentration of 200 or 300 μg ml^-1^. Cells were harvested by centrifugation for 10 min at 14,300 × *g* and 4°C. The cell pellet was resuspended in 500 μl cell lysis buffer (10 mM Tris-HCl pH 7.5, 10 mM MgCl_2_, 30 mM NH_4_Cl, 100 or 150 μg ml^-1^ chloramphenicol) and either mixed with an equal volume of glass beads (300 μm in diameter) and vortexed for 5 min at 4°C or immediately frozen in liquid nitrogen (Bronowski et al., 2014). Suspensions with glass beads were centrifuged for 10 min at 6,400 × *g* at 4°C. The supernatant was collected, centrifuged for 3 min at 17,649 × *g* at 4°C and immediately applied to sucrose density centrifugation or stored at -70°C. Frozen suspensions were thawed in an ice bath, frozen again in liquid nitrogen and stored at -70°C.

The protocol for the sucrose density centrifugation was adapted from Jiang et al. (2007). An A_260_ of 8 of the cleared cell lysate was loaded onto a gradient of 5–45% sucrose in buffer (10 mM Tris-HCl pH 7.5, 10 mM MgCl_2_, 100 mM NH_4_Cl). Ultracentrifugation was carried out for 4 h at 4°C and 253,483 × *g* in a Beckman SW-41Ti rotor. Fractions of the gradient were collected using an UA-6 system (Teledyne ISCO) with continuous monitoring at A_254_.

### Numerical and Statistical Analysis of Ribosome Profiles

Ribosome profiles were scanned, traced in CorelDraw to increase contrast and xy-coordinates were extracted using DataThief III (Pascoe et al., 2015). To increase the reliability of calculations, in addition to peak values we calculated the Xgrad-values using a code (XSpan) written in VisualBasic. The code is available to interested readers upon request. The program ‘XSpan’ places the largest rectangular surface with a predefined width (= X) under individual peaks of a given curve and then calculates the Xgrad-values, which are height (H) and area (A) of the surfaces. XSpan can also extrapolate clipped curves (e.g., when a maximum value exceeds the measurement range) by fitting a cubic function such that it tangents the two flanks of a clipped peak. This option was utilized in this study to obtain the 70S heights (H). The Xgrad-value H is the highest amplitude (absorbance) measured for the surface of predefined width describing fractions of the analyzed sedimentation gradient. X, the width of the rectangular surfaces, was selected such that it covers approximately 1.2% of the gradient fractions analyzed. Statistical analysis was performed using the H values only. For each profile, the H values of the 30S and 50S subunit peaks were normalized to the H values of the first polysome peak and statistical significance was calculated using the paired Student’s *t*-test. Statistical significance was assumed with *p*-values below 0.05.

### PCR Screening, DNA Sequencing, and Sequence Alignment of *fic* Genes

Prevalence of TA genes was surveyed among *C. fetus* isolates via PCR using chromosomal DNA as template. We applied primer pairs 1/2 for *fic1*, 26/27 for *fic2*, 5/6 for *fic3*, 7/8 for *fic4*, and 9/10 for *fti3* (Supplementary Table S2). Sequencing of *fic2* amplicons from *C. fetus* subsp. *venerealis* strains V9, V20, V32, V60, V62, and V69 was performed with primers 28/29.

### Phylogenetic Analysis of *Campylobacter* spp. Fic Proteins

The conserved Fido motif sequence HPFXXGNXR and full length Fic1-4 of *C. fetus* subsp. *venerealis* 84-112 were used in BlastP analysis to identify Fido proteins in whole genomes of *Campylobacter* species (if possible, finished whole genomes, if not available, genomes with low scaffold numbers were used). BlastP analysis was also performed with full-length Fics and the Fic2 specific motif sequence [HPFREGNTRTIA] under exclusion of epsilon-proteobacteria to screen for hits outside this class. Selected *Campylobacter* proteins, selected Fic reference proteins, as well as other bacteria from the urogenital tract identified by the BlastP were then used to generate the phylogeny tree. Retrieved proteins were aligned with MEGA6.06 using the BLOSUM matrix. The Neighbor joining tree was constructed with MEGA6.06 (Tamura et al., 2013). The tree was rooted to the translated ORF of housekeeping gene *glnA* of *C. fetus* subsp. *venerealis* 84-112. Protein accession numbers are listed in Supplementary Table S4.

## Results

### FIC Domain Proteins of *C. fetus* subsp. *venerealis* 84-112

**FIGURE 1:**
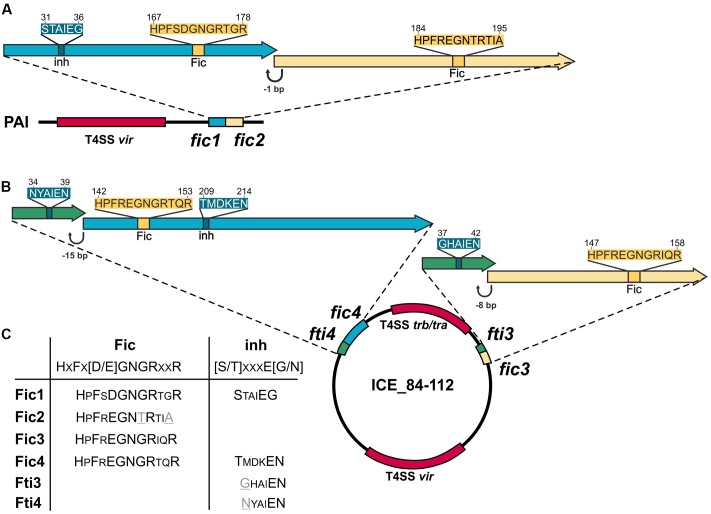
Genetic organization of *Campylobacter fetus* subsp. *venerealis* 84-112 encoded *fic* genes. **(A)** Tandem *fic1* and *fic2* genes on the chromosomal pathogenicity island (PAI) overlap by 1 bp. **(B)** The two loci carried by the extrachromosomal element ICE 84-112 include *fti3* and *fic3*, which overlap by 8 bp, and *fti4* and *fic4* which overlap by 15 bp. *fic* gene clusters are located near loci encoding type IV secretion systems (T4SS), shown in red. Genes encoding solely a Fic motif are drawn as yellow arrows. Genes encoding an additional inhibitory motif (inh) are blue. Genes with only the inhibitory motifs are shown in green. **(C)** Conserved residues of each Fic protein are compared with signature Fic and inh motifs. Non-canonical amino acids in the Fic2 motif and Fic4 inh are indicated in gray and underlined.

*fic1* and *fic2* genes (**Figure 1A**) are chromosomally encoded and form part of a pathogenicity island (PAI) that harbors additionally a functional T4SS (Gorkiewicz et al., 2010). *C. fetus* subsp. *venerealis* 84-112 also carries extra-chromosomal DNA with features of an integrative conjugative element (ICE_84-112) (Kienesberger et al., 2014). Two additional *fic* gene homologs, *fic3* and *fic4*, were identified on the ICE (**Figure 1B**). Residues of the Fido superfamily core motif that enable FIC-containing enzymes to act as AMP transferases have been defined as HxFx(D/E)GNGRxxR (Kinch et al., 2009; Worby et al., 2009; Xiao et al., 2010; Engel et al., 2012). Fic1, Fic3, and Fic4 contain the complete signature of invariant residues [protein accession numbers CDF65254.1 (Fic1); CDF65920.1 (Fic3); CDF65967.1 (Fic4)]. In contrast, in Fic2 (CDF65253.1), the second conserved glycine at position 191 is replaced with threonine and the final arginine of the signature motif (R195A) is absent, suggesting that Fic2 does not have adenylylation activity. Fic1 and Fic4 also contain a conserved inhibitory motif (S/T)xxxE(G/N), which was shown to suppress adenylylation in well-studied systems (Harms et al., 2016b) (**Figure 1C**). Fic proteins containing this inhibitory helix (inh) are classified depending on whether the inh is part of the FIC fold as an N-terminal helix (class II) or a C-terminal helix (class III) (Engel et al., 2012). Fic1 thus belongs to class II, and Fic4 to class III. Class I Fic proteins do not contain the inhibitory motif themselves, but have an interaction partner that provides the inh *in trans*. Fic2 and Fic3 lack a motif with this overall consensus, thus they belong to class I. Fic1 may act as antitoxin for the degenerated toxin Fic2. Alternatively, mutations of the core motif in Fic2 may have altered enzyme activity and thus have bypassed the need for an inh motif. We also note that the 78 amino acid ORF (protein accession number CDF65919.1) upstream and partially overlapping *fic3* includes residues GHAIEN, which might provide the invariable glutamate (Engel et al., 2012; Goepfert et al., 2013) of a poorly conserved inhibitory motif (**Figure 1B**). Presence of a 48% identical homolog, *fti4* (protein accession number CDF65966.1), upstream and partially overlapping *fic4* strengthens the hypothesis that each ORF encodes a small interacting protein to control the cognate FIC enzyme.

### Protein Structure Prediction

**FIGURE 2:**
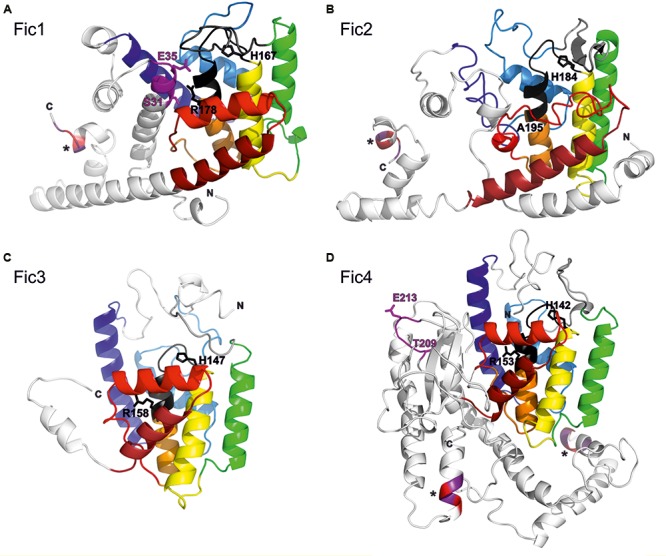
Structural models of *C. fetus* subsp. *venerealis* Fic proteins. The structure of each Fic protein **(A–D)** was predicted with Phyre2. The rainbow colors from blue (N-termini; N) to red (C-termini; C) illustrate the core FIC domain secondary structure topology, as defined in Kinch et al. (2009). Black designates each FIC core motif. Pink specifies the inhibitory motifs (Fic1 and Fic4). Key residues within these motifs are numbered. Protein extensions outside the Fic fold are shown in white. Asterisk designates putative C-terminal translocation signals containing conserved KEKE residues.

Alignment of the predicted proteins shows strong conservation of the Fic core motif but low general similarity (not shown). A structure prediction was performed with the Phyre2 server using templates listed in Supplementary Table S4. All *C. fetus* subsp. *venerealis* homologs are predicted to share a similar FIC domain fold (**Figures 2A–D**). The set of common α-helices are colored from blue (N-terminal) to red (C-terminal), according to the core FIC domain secondary structure topology (Kinch et al., 2009). The predicted active site loops with the conserved core motif including the catalytic histidine are highlighted in black. Fic proteins typically carry a β hairpin close to the active site (gray). This structure, also called “the flap,” constitutes the major target-protein docking site (Kinch et al., 2009; Xiao et al., 2010; Palanivelu et al., 2011; Garcia-Pino et al., 2014). The inhibitory motifs of Fic1 and Fic4, expected to prevent the adenylylation reaction by active site obstruction, are shown in pink. The remaining protein structure outside of each FIC core domain is shown in white. One additional shared feature we noted is the conserved KEKE motif (asterisks) at the C-termini of Fic1 and Fic2 that is reiterated in Fic4, once internally, and again at the C-terminus. Bacterial effector proteins secreted via a given T4SS typically display a short C-terminal stretch of conserved residues that mediates their specific recognition by the transfer machinery (Zechner et al., 2012; Christie et al., 2014). The conserved KEKE motif may represent such a dedicated translocation signal, but this has not been validated experimentally.

### Fic1 and Fic2 Form a Functional TA Module

**FIGURE 3:**
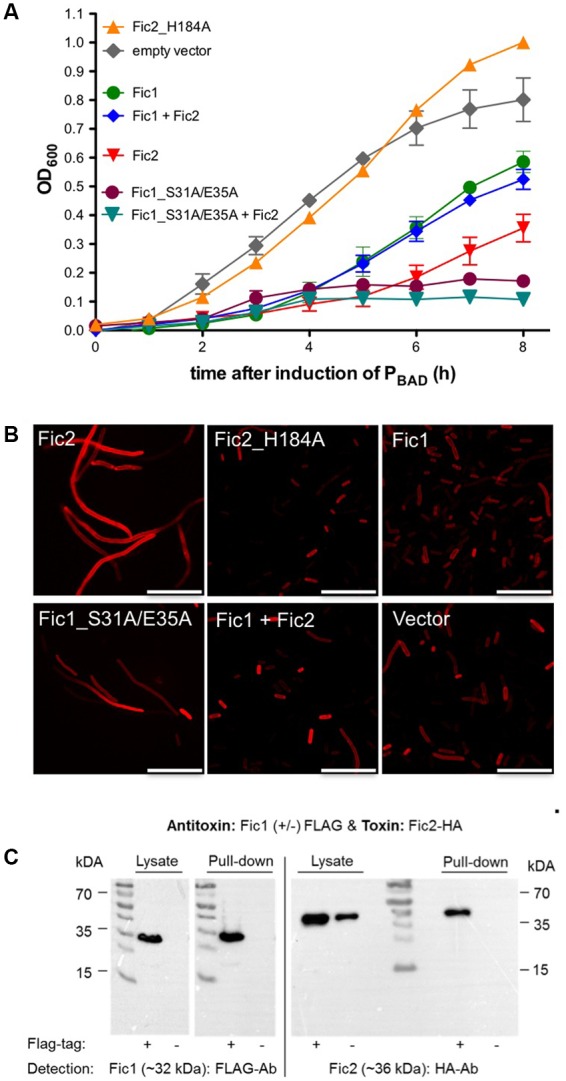
Toxicity of Fic2 in *Escherichia coli* is neutralized by coexpression of Fic1. **(A)** Comparison of growth profiles of *E. coli* expressing *fic1* or *fic2* wild type alleles, mutant alleles, respective combinations, or the empty vector control. Results are mean values of OD_600_ ± standard deviation of three independent experiments. **(B)** Confocal microscopy of Nile red stained *E. coli* expressing indicated *fic* proteins or derivatives alone or in combination. Vector control (lower right panel), scale bars (25 μm). **(C)** Co-immunoprecipitation of Fic1 (32 kDa) and Fic2 (36 kDa). Either FLAG-tagged (+) or native (–) Fic1 were expressed in *E. coli* together with HA-tagged Fic2 and incubated with FLAG-affinity beads. Cell lysates and elution fractions (pull-down) were loaded on gels and proteins were detected by Western analysis using anti-FLAG- or anti-HA-antibodies (Ab).

To gain insights to the function of the *C. fetus* proteins we expressed these in a heterologous bacterial host and asked whether the *fic1*-*fic2* module acts as a TA system. In that case, the inhibitory domain of Fic1 would be required to act both intra- and intermolecularly to regulate the enzymatic activity of Fic1 and Fic2. The *fic* genes of *C. fetus* were placed under transcriptional control of the P_BAD_ promoter and their effects on growth of *E. coli* were investigated. Shifting *E. coli* cells from LB broth with glucose to medium containing arabinose induced synthesis of the Fic proteins and culture density was monitored over time. Induction of *fic2* expression delayed growth of *E. coli* severely compared to the vector control, demonstrating that Fic2 is toxic despite its degenerate core motif (**Figure 3A**). Exchange of the catalytic histidine in variant Fic2_H184A eliminated toxicity and allowed the host to grow comparably to the vector control strain. Loss of phenotype could occur either because the histidine is indeed important to the activity of the enzyme, as predicted, or because the mutant variant is unstable. To exclude the latter possibility we purified the mutated protein and verified its stability during overexpression in *E. coli* and in isolated form (not shown). *E. coli* expressing *fic1* displayed logarithmic growth, but culture densities obtained after 8 h were lower than cells carrying the empty vector. To examine the role of the conserved inh of Fic1, key residues Ser31 and Glu35 were exchanged for alanine. The substitution apparently disrupted the protective function of this motif, since expression of Fic1_S31A/E35A was incompatible with cell growth. Cells were rescued from Fic2-induced growth arrest by co-expression of wild type Fic1, suggesting that Fic1 can act as an antitoxin for Fic2. The importance of the inh module in toxin neutralization was again shown when co-expression of Fic1_S31A/E35A and Fic2 arrested growth fully. The data imply that Fic1 catalyzes an activity detrimental to bacterial growth, but which is normally blocked intramolecularly by the protein’s inh helix. Moreover, the bacterial cytotoxicity of Fic2 depends on the enzyme core motif and is neutralized by antitoxin Fic1.

To characterize the proposed toxin–antitoxin activities, we next compared the impact of Fic protein production on cellular morphology. Fic2 alone caused an extreme filamentous phenotype (**Figure 3B**) via a mechanism requiring the catalytic histidine since; by comparison, cells expressing Fic2H184A were similar to wild type. Cells expressing Fic1 appeared normal but formed filaments when the Fic1 inh motif was mutated. Coexpression of wild type Fic1 reversed the filamentous phenotype caused by Fic2 consistent with the neutralization observed during growth (**Figure 3A**).

Antitoxins similar to the PhD-Doc paradigm frequently inactivate the toxin by forming a stable complex. We asked whether inactivation of Fic2 toxicity by Fic1 might involve binding of the two proteins. Codons for a FLAG epitope were added to *fic1* and the hemagglutinin (HA) tag was added to *fic2*. Lysates of *E. coli* cells expressing both fusion proteins were incubated with FLAG-affinity beads. After elution of bound proteins, lysates and eluates were analyzed by western immunoblotting (**Figure 3C**). Anti-FLAG antibodies confirmed the presence of FLAG-tagged Fic1 in cell lysates and the absence of signal in control samples expressing native Fic1. Antibody to HA detected Fic2-HA fusion protein in the same cell lysates. HA signal in the pull down fraction indicated retention of Fic2 by Fic1. The specificity of this interaction was confirmed by the absence of signal when partner protein Fic1 lacked the FLAG epitope. These properties indicate that Fic1 and Fic2 of *C. fetus* subsp. *venerealis* 84-112 form a functional toxin–antitoxin system. Given that the enzymatic activity of antitoxin Fic1 is autoregulated via inh, this protein exhibits a mode of concomitant intra- and intermolecular- toxin neutralization novel for bacterial Fic proteins.

### Fti3 Acts as an Antitoxin for Fic3

**FIGURE 4:**
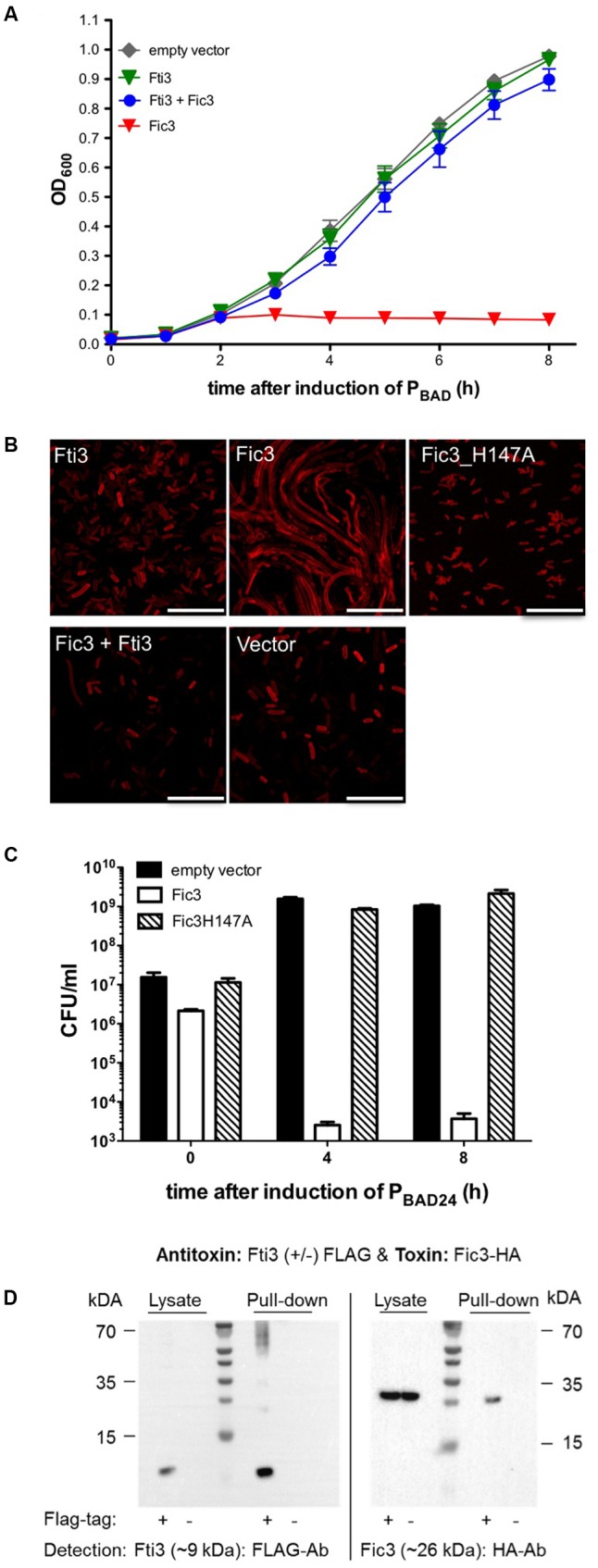
Toxicity of Fic3 in *E. coli* is neutralized by coexpression of Fti3. **(A)** Growth profiles of *E. coli* expressing *fti3* or *fic3* or the combinations compared to the empty vector control. Results are mean values of OD_600_ ± standard deviation of three independent experiments. **(B)** Confocal microscopy of Nile red stained *E. coli* expressing indicated *fti* or *fic* genes or derivatives, respectively, alone or in combination. Vector control (lower right panel), scale bars (25 μm). **(C)** Colonies formed (CFU per ml) 0, 4, and 8 h post-induction for *E. coli* expressing *fic3*, *fic3*_H147A or the vector control. Results are mean values of three independent experiments. **(D)** Co-immunoprecipitation of Fti3 (9 kDa) and Fic3 (26 kDa). Either FLAG-tagged (+) or native (–) Fti3 were expressed in *E. coli* together with HA-tagged Fic3 and incubated with FLAG-affinity beads. Cell lysates and elution fractions (pull-down) were loaded on gels and proteins were detected by Western analysis using indicated anti-FLAG- or anti-HA-antibodies (Ab).

Fic3, like Fic2, carries the enzyme core motif but lacks an inh motif (**Figure 1**). Similar to the result of *fic2* expression, *E. coli* carrying *fic3* failed to grow under inducing conditions (**Figure 4A**). However, dual expression of *fic3* and its neighboring gene encoding the putative inhibitor protein restored *E. coli* growth completely. *E. coli* cells expressing the inhibitor alone grew indistinguishably from cells carrying the vector control. Since, this protein acts as an antitoxin for Fic3 we named the gene *fti3* (Fic toxin inhibitor 3). Microscopy of the toxin/antitoxin expressing *E. coli* revealed that inhibitor protein alone had no impact on cell morphology (**Figure 4B**). By contrast we observed extreme filamentation due to Fic3 that could be reversed by either mutation of the catalytic histidine in variant Fic3H147A, or co-expression of wild type Fic3 with Fti3. To assess the viability of cells expressing Fic3, samples of a culture before and after 4 or 8 h of arabinose-induced expression were plated on LB agar without arabinose. Viability of the culture dropped by several orders of magnitude after Fic3 induction (**Figure 4C**). By contrast cells producing the non-toxic variant Fic3H147A exhibited similar viability as the vector control strain. Direct interaction between Fti3 and toxin Fic3 was tested following coexpression of FLAG-tagged Fti3 and HA-tagged Fic3. FLAG-tagged Fti3 from the cell lysate bound the affinity matrix and specifically retained Fic3-HA in the pull down reaction (**Figure 4D**). No retention of Fic3 was detected when Fti3 lacked the FLAG epitope. We conclude that Fti3-Fic3 form another TA module on the extrachromosomal ICE in addition to the chromosomal system *fic1-fic2*.

### Putative Antitoxin Fti4 Interacts with Fic4

**FIGURE 5:**
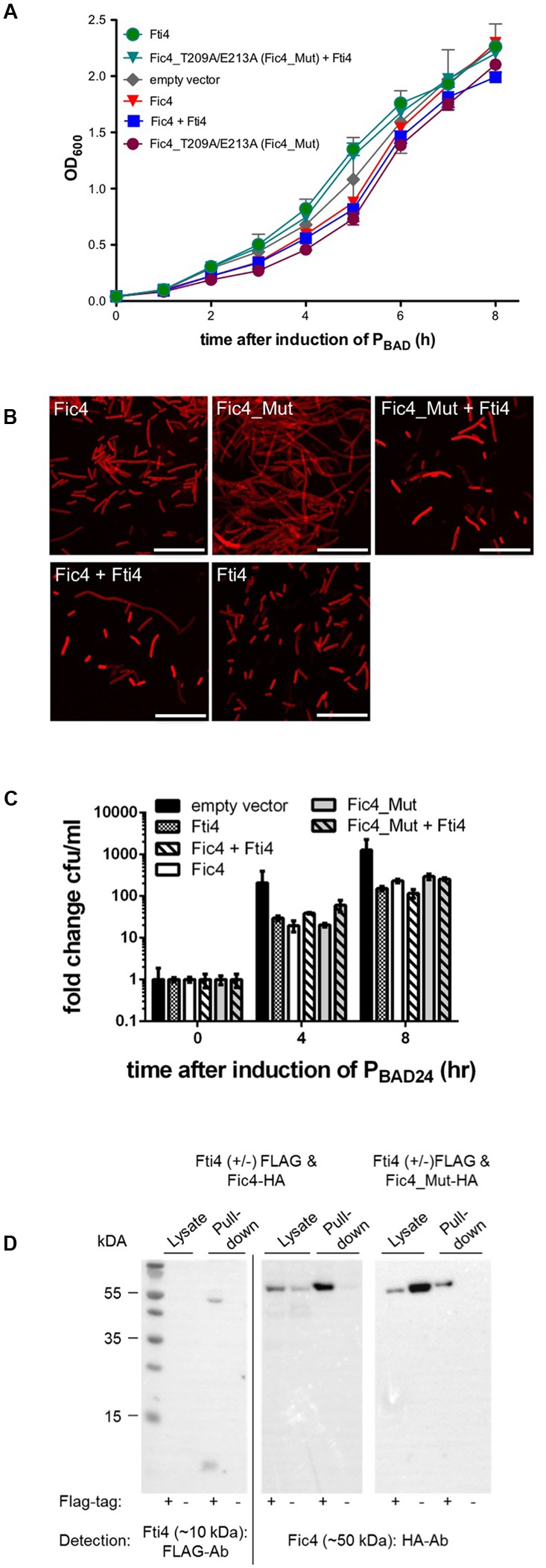
Fti4 interacts with the non-toxic Fic4. **(A)** Growth profiles of *E. coli* expressing indicated *fti* and *fic* genes, or derivatives, alone or in combination. Results are mean values of OD_600_ ± standard deviation of three independent experiments. **(B)** Confocal microscopy of Nile red stained cells cultured in **(A)** as indicated. Fic4_Mut refers to Fic4_T209A/E213A. Scale bars (25 μm). **(C)** Colonies formed (CFU per ml) 0, 4, and 8 h post-induction for *E. coli* expressing *fic4* and *fic4*_209A/E213A with or without Fti4, or vector control. **(D)** Co-immunoprecipitation of Fti4 (10 kDa) and Fic4 (50 kDa). Either FLAG-tagged (+) or native (–) Fti4 were expressed in *E. coli* together with HA-tagged Fic4 and incubated with FLAG-affinity beads. Cell lysates and elution fractions (pull-down) were loaded on gels and proteins were detected by Western analysis using indicated anti-FLAG- or anti-HA-antibodies (Ab).

Expression of *fic4* for up to 8 h had no effect on cell growth (**Figures 5A,C**) and affected cell morphology only mildly (**Figure 5B**). To test whether the protein’s inhibitory motif was suppressing the predicted enzyme activity, residues Thr209 and Glu213 were exchanged for alanine. Expression of the mutant variant was compatible with normal growth comparable to cells expressing wild type Fic4 or the vector control (**Figure 5A**) but a filamentous phenotype was observed upon Fic4_T209A/E213A-HA expression (**Figure 5B**). Since cells expressing wild type Fic4 are phenotypically normal under these conditions we used the mutant variant to test for a possible antitoxin activity for the adjacent ORF, Fti4. Coexpression of Fti4 and mutant Fic4 lessened filamentation substantially (**Figure 5B**) but had no impact on growth or survival (**Figures 5A,C**).

To test whether Fti4 and Fic4 physically interact, fusion proteins with epitope tags were created and simultaneously produced in *E. coli* as described above. FLAG-Fti4 (∼10 kDa) was not directly detectable in the cell lysates but was visible after enrichment of the protein on the affinity matrix (**Figure 5D**). Fic4-HA was detected in lysates of both test and control strains. Fic4-HA was also retained on the FLAG affinity beads in a manner dependent on FLAG-Fti4. Since the functional tests described above showed phenotypes for Fti4 only when combined with the mutant derivative of Fic4, we also assayed for protein binding using the mutant allele. Similar to wild-type Fic4, co-retention of Fic4_T209A/E213A by FLAG-Fti4 was observed (**Figure 5D**, right panel).

In summary, some of the observed characteristics of Fic4 are consistent with the function of a toxin, yet the toxicity of the mutant form was quite mild compared to the inh-deficient Fic1 derivative and the wild type class I proteins Fic2 and Fic3. It is possible that evolution has introduced mutations outside of the Fic4 active site that impair enzyme activity. It is further possible that the surrogate host *E. coli* simply lacks the specific protein targeted by Fic4. Another hypothesis that we could test was to ask whether Fic4 might actually function as an antitoxin for a distinct locus (below).

### Fic2 Toxicity Is Inactivated *in Trans*

**FIGURE 6:**
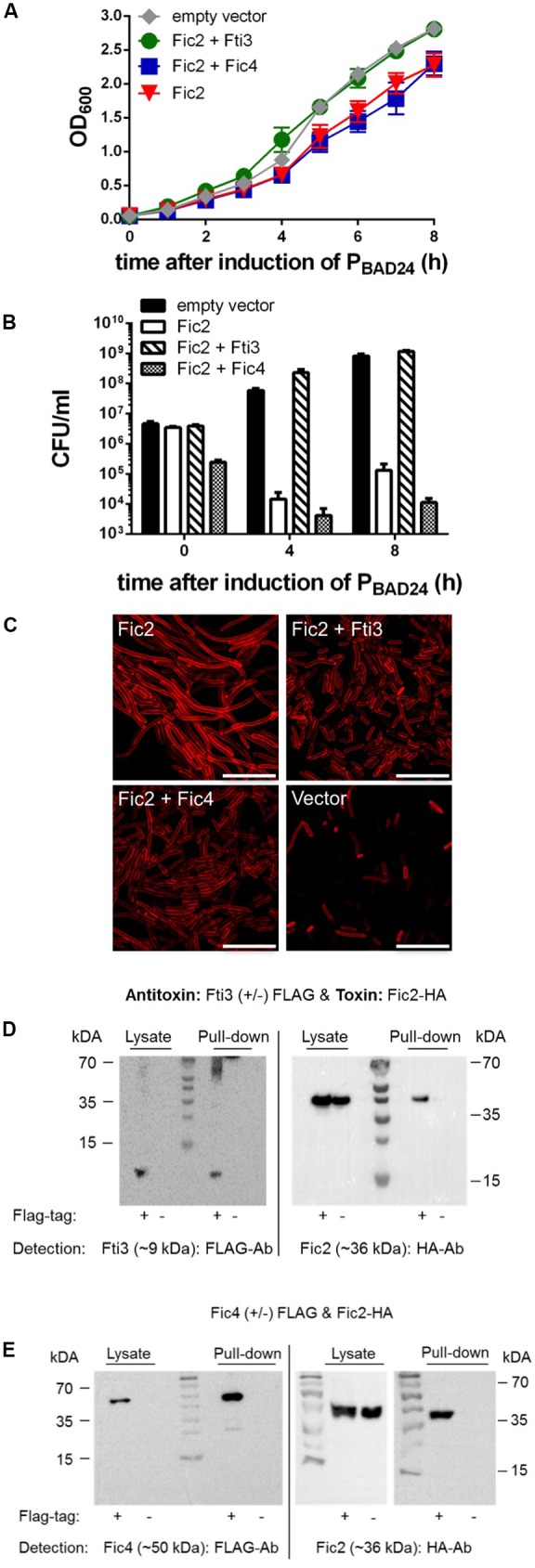
Fic2 interacts with Fti3 and Fic4 and Fic2 toxicity is neutralized by Fti3. Growth profiles over time **(A)** and corresponding colonies formed (CFU per ml) **(B)** at 0, 4, and 8 h post-induction of *E. coli* expressing *fic2* alone or in combination with either *fti3* or *fic4*. Results are mean values of OD_600_ or CFU/ml ± standard deviation of three independent experiments. **(C)** Confocal microscopy of Nile red stained *E. coli* expressing indicated *fic2* alone or in combination with *fti3* or *fic4*. Vector control (lower right panel), scale bars (25 μm). **(D)** Co-immunoprecipitation of Fti3 (9 kDa) and Fic2 (36 kDa) and **(E)** Fic4 (50 kDa) and Fic2 (36 kDa). Either FLAG-tagged (+) or native (–) Fti3 **(D)** or Fic4 ± FLAG **(E)** were expressed in *E. coli* together with HA-tagged Fic2 and incubated with FLAG-affinity beads. Cell lysates and elution fractions (pull-down) were loaded on gels and proteins were detected by Western analysis using indicated anti-FLAG- or anti-HA-antibodies (Ab).

To explore potential *in trans* interactions involving components of the distinct systems each toxin was expressed pairwise with every putative antitoxin. We found that the ICE_84-112 encoded antitoxin Fti3 reversed the growth defect caused by toxin Fic2 (**Figure 6A**). In contrast co-expression of Fic4 with Fic2 had no effect. Neutralization of Fic2 toxicity by Fti3 was confirmed by plating samples of the induced cultures (**Figure 6B**). Cells survived 4 and 8 h of Fic2 expression when co-expressing Fti3, but Fic4 was not able to counteract the toxicity of Fic2. Functional interaction between Fic2 and Fti3 was also supported by the normalized morphology of cells following co-expression compared to the filamentous phenotype caused by Fic2 alone (**Figure 6C**). A partial reversal of the Fic2-induced filamentation was observed with Fic4.

To test for direct binding interactions between the protein pairs, pull-down assays were performed with cells expressing Fic2-HA with either FLAG-tagged Fti3 or FLAG-tagged Fic4. Consistent with the functional results shown above, Fti3 was able to retain Fic2 (**Figure 6D**). Remarkably, although Fic4 showed little antitoxin activity for Fic2, a complex of these proteins was detected nonetheless (**Figure 6E**).

To complete the analyses for toxin Fic2, the same tests were performed for the last putative antitoxin Fti4. No neutralizing activity by Fti4 was observed during cellular growth or by monitoring cell morphology. The pull down assay combining FLAG-tagged Fti4 with Fic2-HA also failed to detect interaction between these proteins (all data not shown). Given that Fti4 is 48% identical to Fti3, the lack of activity observed for Fti4 shows that the ability of antitoxin Fti3 to inactivate Fic2 is specific. As a final specificity check we also tested whether the toxic form of Fic1 (Fic1_S31A/E35A) was affected by co-production of either Fti3 or Fti4. No reversion of the poor growth, reduced survival or filamentous phenotypes caused by Fic1_S31A/E35A was observed (data not shown).

The sum of these data demonstrate that the bacterial growth phenotype caused by Fic2 is counteracted by the *cis* encoded antitoxin, Fic1, and independently by Fti3 *in trans*. Fic4 partially reversed the toxic effect of Fic2 in *E. coli*. Both the *cis* acting antitoxin Fic1 and the ICE-encoded proteins Fti3 and Fic4 were shown to bind toxin Fic2. These findings support a model of functional crosstalk occurring between chromosomally and ICE_84-112 encoded Fic proteins that act to control the toxin Fic2.

### Fic4 Interacts with Toxin Fic3

**FIGURE 7:**
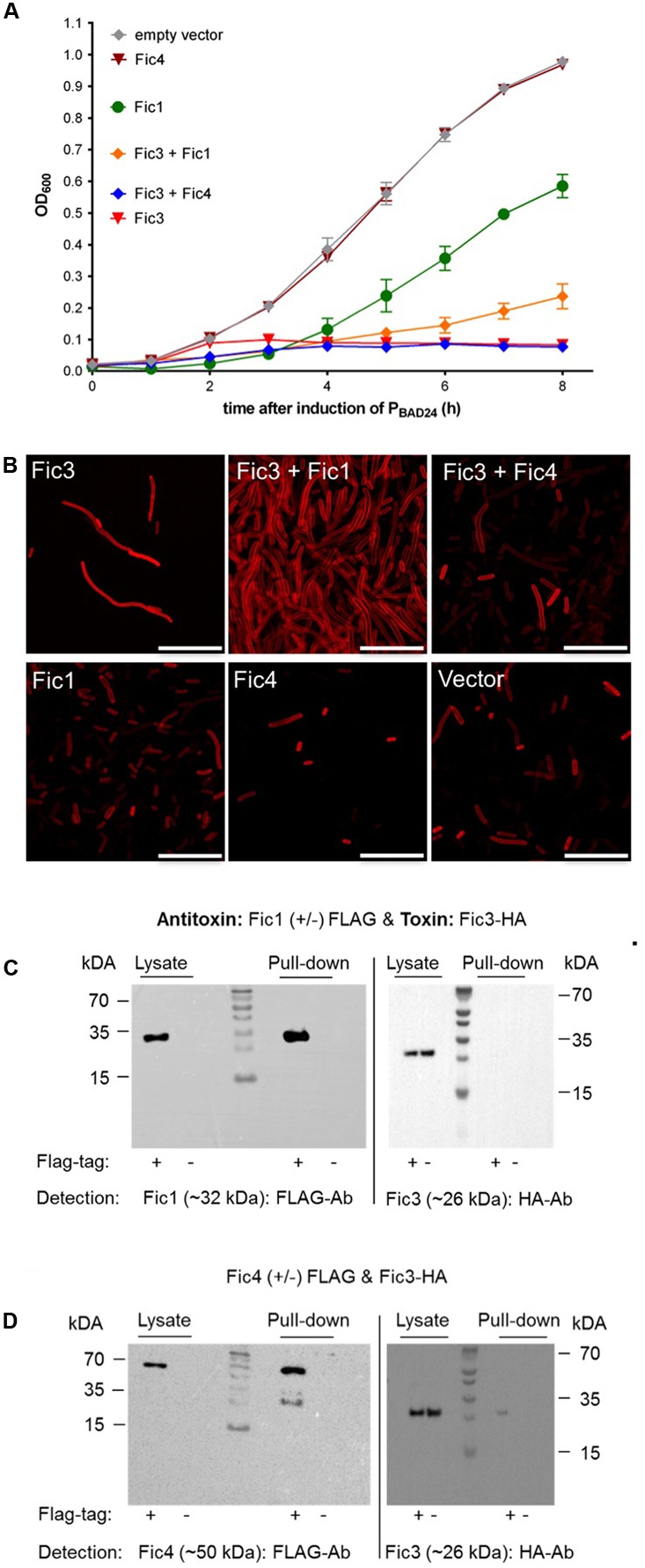
Fic3 toxicity is not relieved by Fic1 or Fic4. **(A)** Growth profiles of *E. coli* expressing *fic3 or fic1* alone or *fic3* in combination with either *fic1* or *fic4*. Results are mean values of OD_600_ ± standard deviation of three independent experiments. **(B)** Confocal microscopy of Nile red stained *E. coli* expressing indicated *fic* proteins alone or in combination. Vector control (lower right panel), scale bars (25 μm). **(C)** Co-immunoprecipitation results for Fic1 (32 kDa) and Fic3 (26 kDa) or **(D)** Fic4 (50 kDA) and Fic3. Either FLAG-tagged (+) or native (–) Fic1 or Fic4 were expressed in *E. coli* together with HA-tagged Fic3 and incubated with FLAG-affinity beads. Cell lysates and elution fractions (pull-down) were loaded on gels and proteins were detected by Western analysis using indicated anti-FLAG- or anti-HA-antibodies (Ab).

To test for potential regulatory crosstalk occurring between Fic3 and antitoxins of the distinct systems, we again performed phenotypic tests following dual expression of each protein pair. Both Fic1 and Fic4 were unable to neutralize the extreme growth phenotype caused by Fic3 (**Figure 7A**). Dual expression of Fic3 with Fic1 did not revert the filamentation induced by Fic3, but partial recovery was apparent upon co-expression of Fic3 with Fic4, suggesting some neutralizing interactions (**Figure 7B**). Consistent with these results the pull-down assay was clearly negative for binding between Fic1 and Fic3 (**Figure 7C**), but a small yield of co-purified Fic3 was detected using FLAG-tagged Fic4 (**Figure 7D**). We performed the same analyses with cells coexpressing Fti4 with Fic3. Again despite its similarity to antitoxin Fti3, Fti4 had no affect on Fic3 toxicity and the proteins failed to bind under these conditions (not shown). In summary, we conclude that Fic3 is effectively neutralized by the *cis* encoded Fti3. Moreover, modest levels of complex formation with trans-acting factor Fic4 may contribute to regulation of this enzyme.

### Fic2 or Fic3 Expression Inhibits Translation in *E. coli*

**FIGURE 8:**
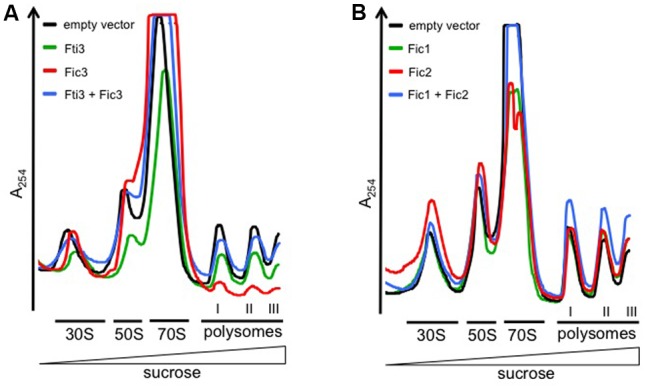
Fic2 and Fic3 disrupt the translational machinery. Comparisons of ribosomal profiles of *E. coli* expressing **(A)** *fti3*, *fic3*, the combination (*fti3* + *fic3*) compared to the vector control, or **(B)** *fic1*, *fic2*, or the combination (*fic1* + *fic2*) compared the vector control. Absorption peaks corresponding to free 30S and 50S ribosomal subunits, 70S ribosomes, and polysomes are designated. The deficit in polysomes relative to free 30S subunits provoked by *fic2* and *fic3* expression is quantified in **Table 1**.

The identity of specific protein targets modified by Fic enzymes in bacteria is difficult to predict. It is known, however, that the activities of many TA toxins interfere with the translation process either directly, e.g., by cleavage of mRNA or tRNA, or as a downstream effect (Rajashekara et al., 2009; Park et al., 2013). To measure translation in *E. coli* cells expressing *C. fetus* Fic proteins, we performed sucrose gradient centrifugation of cell lysates and recorded polysome profiles (**Figures 8A,B**). In these analyses, the height of the polysome peaks is directly proportional to the translation levels, therefore translation defects can be faithfully detected by the reduction of polysome peaks. Moreover, because free ribosomal subunits, 70S monosomes and polysomes can be resolved, changes in the ratios between these different ribosomal (sub-) complexes can give additional information on the type of defect causing reduced translation. We recorded profiles from *fic*-expressing *E. coli* cells and compared them to those of the vector control strain. The signal corresponding to ribosomal subunits and translating ribosomes is indicated for each gradient. To make *fic*-dependent shifts in the relative abundance of these populations more apparent, profiles from different expressing strains were overlaid in the figure. Expression of Fic3 inhibited translation severely, as obvious from the massive reduction of polysome levels (**Figure 8A**, red trace; **Table 1**) compared to the vector control strain (black trace) or cells expressing just antitoxin Fti3 (green trace). Concomitantly, a strong increase of the 70S peak was detected, suggesting that ribosomal subunits are competent for joining into 70S ribosomes, but fail to enter into translation. We conclude that Fic3 blocks a step after subunit joining but before translation elongation. Dual expression of Fic3 and antitoxin Fti3 (blue trace) largely restored translation to normal levels. Moreover, the abnormally high 70S peak observed upon Fic3 overexpression was partially reduced upon Fti3 co-expression.

**Table 1:** Numerical and statistical analysis of ribosomal subunit peaks.

		Ratio subunits vs. polysome (I)		
		30S	50S	70S^∗^
^A^Set 1	Vector	1.06	1.42	6.76
	Fic1	1.08	1.52	3.15
	Fic2	1.47	1.70	2.64
	Fic1 + Fic2	0.86	1.26	3.07
^B^Set 2	Vector	1.06	1.40	3.21
	Fti3	1.16	1.34	3.38
	Fic3	1.94	nd	11.84
	Fti3 + Fic3	1.10	1.67	1.67
		Vector	Fic2	*p*-Values^∗∗^
Statistic (*n* = 7)	30S	1.10 ± 0.02	1.50 ± 0.19	0.0005
	50S	1.49 ± 0.11	2.03 ± 0.38	0.0137

^A,B^*see **Figure 8**; Numbers represent ratios of 30S or 50S subunit versus first polysome peak (I) values. Numbers were calculated based on XSpan generated “Heights” (see section “Materials and Methods”); nd, 50S peaks are not separated enough from 70S peaks for numerical analysis with XSpan; ^∗^Height was generated by XSpan after extrapolation of clipped curves. ^∗∗^*p*-Values were calculated from seven profiles using the paired Student’s *t*-test*.

Fic2 expression also had a mild inhibitory effect on translation, as obvious from an accumulation of free 30S and 50S ribosomal subunits relative to the amount of 70S ribosomes and polysomes (**Figure 8B**). We compared the free subunit accumulation relative to the polysome abundance in multiple independent experiments (*n* = 7) and determined a quantitatively significant increase (**Table 1**). Notably, in contrast to Fic3 expression, Fic2 expression did not result in increased 70S levels, and even reduced 70S amounts compared to the vector control. Reduced 70S and increased free subunit levels are indicative of inefficient subunit joining. *E. coli* cells expressing antitoxin Fic1 (green trace) showed no significant variation in the ratio of free subunits versus polysomes compared to profiles from the vector control strain (**Figure 8B** and **Table 1**). The relative abundance of 70S species was mildly reduced in *fic1*-expressing vs. vector control cells however (**Table 1**), consistent with the observation that Fic1 expression slightly inhibits cell growth (**Figure 3**). In line with the neutralizing activity observed in our previous functional tests, simultaneous expression of antitoxin Fic1 with Fic2 (blue trace) resulted in a profile similar to the empty vector control.

### Prevalence of *fic* Genes within *C. fetus* Strains

The sum of our findings suggests that the *C. fetus fic* genes act as TA systems. In that case the loci should be well-conserved within the species. We used PCR to survey the prevalence of the *fic* genes and *fti3* in 102 *C. fetus* isolates from geographically and ecologically diverse sources (summary in **Table 2**; detailed information in Supplementary Table S5). All of the *C. fetus* subsp. *venerealis* strains (*n* = 62) were positive for *fic1* and 59 out of 62 (95%) were positive for *fic2*. This finding is consistent with genetic linkage of the Fic2 toxin to the Fic1 antitoxin. Sequence analysis of full-length *fic2* amplicons randomly selected from our strain collection (*n* = 6) showed complete conservation for this subspecies (data not shown). In contrast, only 5 out of 40 (12.5%) *C. fetus* subsp. *fetus* strains harbor *fic1* and only two carry the *fic2* gene, whereby strain *C. fetus* subsp. *fetus* 98/v445 (F37) lacks the corresponding *fic1* antitoxin gene. Sequence analysis of this solitary *fic2* allele revealed 36 nucleotide changes, corresponding to 13 amino acid substitutions. Expression of the F37 *fic2* gene in *E. coli* confirmed that the mutated toxin is functionally impaired (data not shown). The *fic3* and *fic4* genes were detected exclusively in *C. fetus* subsp. *venerealis*, *fic3* in 11.3% (7/62) and *fic4* in 4.8% (3/62) of the isolates. Gene *fti3* shows higher abundance: 59.7% (37 of 62) *C. fetus* subsp. *venerealis* isolates and four *C. fetus* subsp. *fetus* isolates (4/40) carry the gene. Consistent with the predicted selective pressure for co-existence, all strains positive for *fic3* additionally encode the corresponding antitoxin Fti3. Moreover the high prevalence of *fic2* in *C. fetus* subsp. *venerealis* may select for stable maintenance of *fti3* even in the absence of the cognate toxin *fic3*.

**Table 2:** Prevalence of *fic* genes in *C. fetus* subspecies.

		Chromosomal			ICE_84-112			
	#	*fic1*	*fic2*	*fic1*+*fic2*	*fic3*	*fic4*	*fti3*	*fti3*+*fic3*
*Cff^a^*	40	5	2	1	0	0	4	0
*Cfv^a^*	62	62	59	59	7	3	37	7

^a^*Cff C. fetus subsp. fetus, Cfv C. fetus* subsp. *Venerealis.*

In summary, we conclude that the presence of a *fic* toxin gene in *C. fetus* is typically linked to carriage of the paired antitoxin gene. The chromosomal TA system is highly conserved in *C. fetus* subsp. *venerealis.* The ICE-associated loci are also unique for *C. fetus* subsp. *venerealis* but are comparatively rare in the strains surveyed.

### Phylogenetic Analysis

**FIGURE 9:**
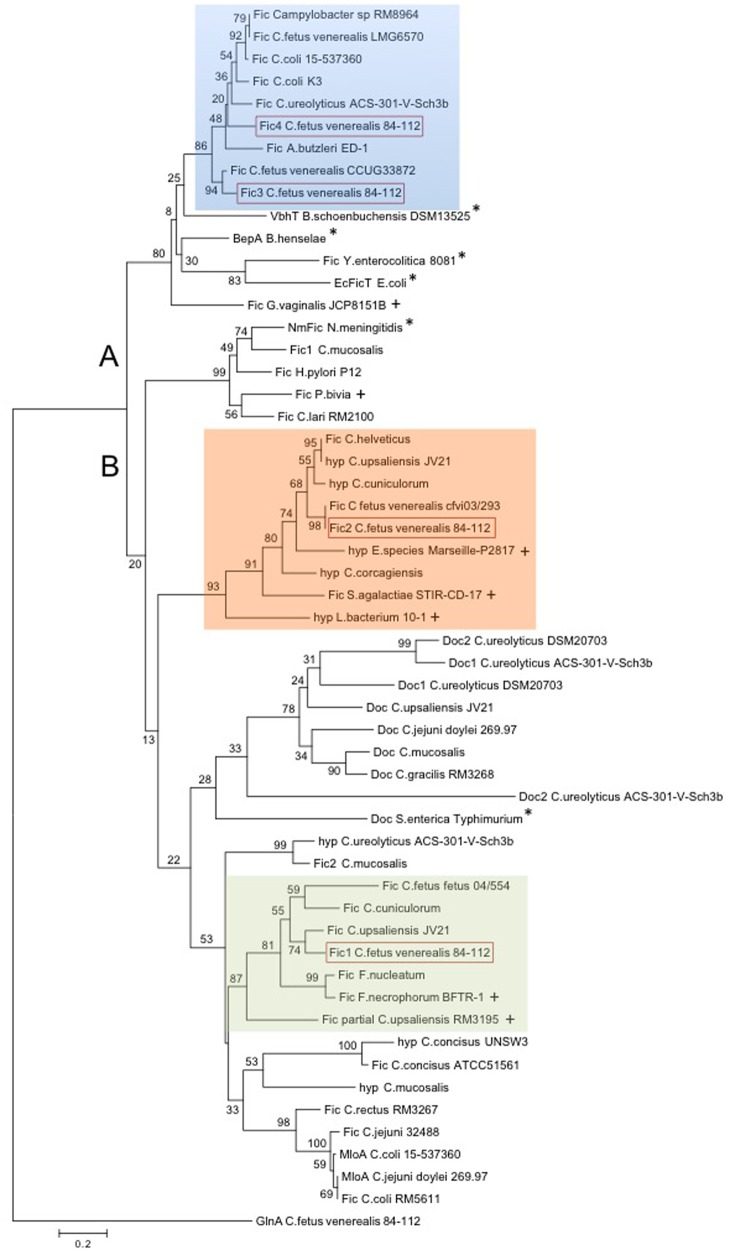
Neighbor joining tree showing phylogenetic relationship of Fido proteins in *Campylobacter* spp. BlastP search of the conserved motif HPFXXGNXR, Fic2 motif HPFREGNTRTIA, and full-length Fic1-Fic4 proteins of *C. fetus* subsp. *venerealis* 84-112 revealed Fido proteins in *Campylobacter* spp., in closely related epsilon-proteobacteria, including *Arcobacter* (*A. butzleri*), *Helicobacter* (*H. pylori*), as well as in more distant species, as indicated. The model template for protein structure prediction of Fic4, VbhT of *B. schoenbuchensis*, as well as other well-described reference Fic proteins were included (asterisk). The Neighbor joining tree contains 54 proteins and is rooted to the *C. fetus* subsp. *venerealis* 84-112 housekeeping protein GlnA. Protein and organism names are shown. Fic1-4 of *C. fetus* subsp. *venerealis* 84-112 are highlighted (red box). Proteins from distant species linked to infertility or abortion are indicated with pluses. Protein accession numbers are listed in Supplementary Table S5. The two obtained clusters (A and B) are indicated. Bootstrap values (1,000 replicates) are shown at the tree nodes. The scale bar represents 0.2 substitutions per amino acid position.

The significant association of these *fic* genes with *C. fetus* subsp. *venerealis* and their relative absence in *C. fetus* subsp. *fetus* led us to next ask whether they are present in other *Campylobacter* species and/or whether they are conserved in bacteria which inhabit the urogenital tract. Using the HPFXXGNXR motif in a BlastP analysis revealed that genomes of several *Campylobacter* species encode from one to four Fido proteins. BlastP analyses were then performed with the full-length *C. fetus* proteins Fic1-4 to identify related Fido proteins from epsilon-proteobacteria or distantly related bacteria. Interestingly, BlastP analysis using full-length Fic2 or the degenerated motif of Fic2 consequently retrieved proteins of bacterial species linked to human fertility complications (Moreno et al., 2016; Pelzer et al., 2017). We used these proteins, related *Campylobacter* proteins and selected reference Fic-proteins (Harms et al., 2016b) to generate the Neighbor joining tree shown in **Figure 9**. The tree architecture placed the proteins in two main branches. Cluster A includes Fic3 and Fic4 of *C. fetus* subsp. *venerealis* 84-112. Reference FIC-domain proteins of *Bartonella*, *Yersinia enterocolitica*, and *E. coli* (marked with asterisks) were also placed in this cluster.

Fic1 and Fic2 are both grouped in the B branch and resolve in separate subclusters. Interestingly, *Fusobacterium* spp. also harbor FIC proteins closely related to Fic1. *Fusobacteria* inhabit mucous membranes of humans and animals and both *Fusobacterium nucleatum* and *Fusobacterium necrophorum* cause abortion in cattle (Kirkbride et al., 1989; Otter, 1996). Moreover *F. nucleatum* causes intra-amniotic inflection and premature delivery in humans and mice (Han et al., 2004; Gauthier et al., 2011). We also note that *C. ureolyticus* ACS-301-V-Sch3b, isolated from the human vaginal tract, encodes a protein related to Fic1 and another related to Fic3 and Fic4 of cluster A.

Fic2 clusters with a hypothetical protein of *Campylobacter upsaliensis* JV21 and proteins of *C. helveticus* and the more recently described novel species *C. cuniculorum* and *C. corgagiensis*. *C. upsaliensis* is a human enteropathogen that typically causes diarrhea, bacteremia and sepsis, but human infection with *C. upsaliensis* has also been associated with spontaneous abortion (Gurgan and Diker, 1994). Moreover, proteins of bacterial species suspected or confirmed to play a role in human fertility and pregnancy outcome cluster in the same branch (indicated with a plus sign).

In conclusion, our findings show conserved FIC protein sequences in a variety of bacteria that either inhabit the urogenital tract of humans and animals or are able to establish pathology in this niche.

## Discussion

**FIGURE 10:**
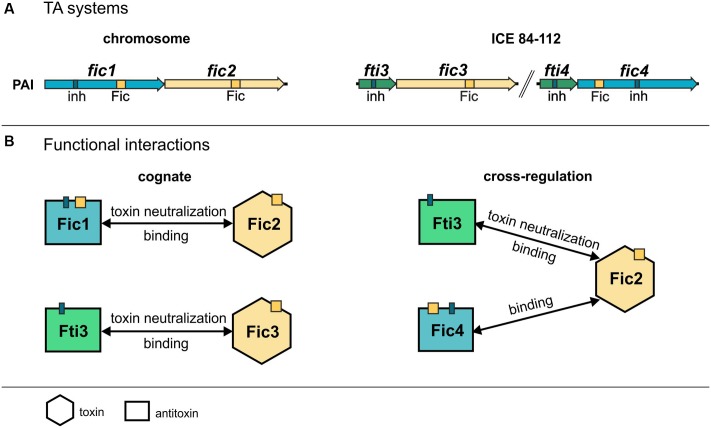
Summary of cognate and non-cognate rescue and interaction data. **(A)** TA modules of *C. fetus* subsp *venerealis* strain 84-112 are depicted. The chromosomal locus encodes AT-Fic1/T-Fic2. The extrachromosomal ICE carries two additional independent modules AT-Fti3/T-Fic3 and the module encoding the putative TA system AT-Fti4/T-Fic4. Conserved active site motifs (Fic) and inhibitory helices (inh) are indicated. **(B)** The functional interaction and physical binding of cognate TA components detected in this study are shown (left). Fti4 binds Fic4 (not shown), but Fic4 has no toxic activity in *E. coli*. Cross-regulation of toxin Fic2 by non-cognate components is shown (right). Two unrelated ICE-encoded proteins, Fic4 and Fti3, bind T-Fic2. Heterologous AT-Fti3 counteracted Fic2 toxicity.

*Campylobacter fetus* subsp. *venerealis* 84-112 expresses a group of Fic proteins that are conserved in various veterinary and human pathogens of the urogenital tract. This study shows that the *fic* modules are prevalent and strongly conserved in *C. fetus* subsp. *venerealis* isolates but generally lacking in *C. fetus* subsp. *fetus.* The data also provide the first experimentally validated examples of TA activity for Fic proteins in *Campylobacter.* Bacterial genomes generally harbor multiple TA modules and it is becoming increasingly clear that the TA-associated toxins perform discrete, multipurpose functions (Ramage et al., 2009; Leplae et al., 2011; Lobato-Marquez et al., 2016; Diaz-Orejas et al., 2017). *C. fetus* subsp. *venerealis* 84-112 carries one functional module on the chromosome. Two additional systems are located on the extra chromosomal element ICE_84-112 (Kienesberger et al., 2014). It has been speculated that TA components encoded by horizontally acquired DNA and chromosomal loci evolve toward functional cooperation (Saavedra De Bast et al., 2008; Makarova et al., 2009). Here, we demonstrate that Fti3 encoded by ICE_84-112 provides immunity for the chromosomal toxin Fic2 in addition to the cognate antitoxin Fic1 (see summary of results, **Figure 10**). We also show that the ICE-encoded Fic4 binds Fic2 and may therefore contribute to toxin regulation by complex formation or sequestration. Oligomerization was shown to play a role in regulating toxin activity for the class III Fic protein NmFic from *Neisseria meningitidis* (Stanger et al., 2016). In that case, activation of the NmFic toxin is blocked by tetramer formation. Control of *C. fetus* toxin Fic2 by Fic1 and possibly Fic4 implies that inhibitory strategies involving heteromeric complexes of different toxins are also possible. The structural similarities of the *C. fetus* Fic proteins may support physical interactions between non-cognate components as shown with the *ccd* and *parD* systems (Smith et al., 2012). Compared to well-studied Fic proteins or paradigm type II TA systems evidence for physical and functional interactions between non-cognate toxin–antitoxin systems is still relatively rare but clearly emerging as shown for *Mycobacterium tuberculosis* (Yang et al., 2010; Zhu et al., 2010). TA systems can also be interconnected through transcriptional regulation, for example positive feedback regulation can allow production of toxins to induce transcription of other TA systems (Kasari et al., 2013). As another example, toxin MqsR of the type II TA system MqsR/MqsA regulates GhoT toxin of a type V TA system via post-transcriptional differential mRNA cleavage. This activity results in a regulatory hierarchy where one TA system controls another (Wang et al., 2013). Wessner et al. (2015) have also established that regulatory crosstalk occurs between modules of type-I and -II TA families in the human pathogen *Enterococcus faecalis*. Taken together these data support the notion that bacteria harboring multiple TA systems may develop a complex hierarchy. Cooperative regulation of their activities would support concerted physiological responses in the cell. Moreover, interplay between TAs may enable bacteria to create heterogeneous populations and survive stress under a wider range of environmental conditions (Fasani and Savageau, 2013).

The ability to enter prolonged dormancy is an important factor in the epidemiology and spread of *Campylobacter* (Rollins and Colwell, 1986; Bronowski et al., 2014). Dormancy requires the bacterial cells to switch from normal activities to a static state (Harms et al., 2016a). Enzymes belonging to the Fido family are well-suited to control this process as they can modify a broad range of cellular proteins post-translationally (Garcia-Pino et al., 2014; Roy and Cherfils, 2015; Harms et al., 2016b). The general lack of similarity outside of the active loop implies that Fic1-4 bind to distinct protein targets. The core motif conserved in Fic1, Fic3, and Fic4 suggests these are competent for adenylylation. The modification reaction catalyzed by Fic2 remains unclear because the core motif deviates from the adenylylation consensus. Regardless of the biochemistry involved, we found that expression of both the canonical Fic3 and the degenerate Fic2 toxins in *E. coli* interferes with translation. The inhibitory effect of Fic3 on translation was very pronounced. Fic3 expressing cells show a very high 70S peak and drastic reduction of polysome levels suggesting that ribosomal subunits are produced and can join, but are incapable of entering into translation. In contrast, Fic2 reduced 70S levels, while the amounts of free ribosomal subunits were increased. These properties are consistent with an early defect that impairs subunit joining. We conclude that Fic2 either directly affects subunit joining, or alternatively, that it inhibits a step of ribosome biogenesis, thereby causing the synthesis of aberrant, joining defective ribosomal subunits. Fic2 might therefore modify an rRNA processing factor or an assembly cofactor. We note with interest that ribosome profiles of cells overexpressing YfjG (RatA), a toxin of the *yfjG-yfjF* operon on the *E. coli* chromosome are similar to those expressing Fic2. YfjG inhibits 70S ribosome association and blocks the translation initiation step (Zhang and Inouye, 2011). Work in other laboratories has shown that mutation or depletion of ribosome assembly GTPases, but also inhibition of translation, is associated with filamentous cell morphology (Karbstein, 2007). These attributes resemble the phenotypes we observed. To better understand the mechanism of bacterial cytotoxicity we are characterizing each of the *C. fetus* subsp. *venerealis* toxins biochemically and structurally.

Although we can generally conclude that the TA systems described here exist in *C. fetus* subsp. *venerealis* 84-112 to control the switch between normal and static metabolic states, details about the biological context of that activity remain unknown. Emerging data from animal models of uropathogenic *E. coli* infection establish that TA systems are important for niche-specific colonization and survival, and a contribution to virulence was described in *Salmonella* Typhimurium (Norton and Mulvey, 2012; De la Cruz et al., 2013; Lobato-Marquez et al., 2015). Thus, presence of multiple Fic proteins in *C. fetus* subsp. *venerealis* may enhance long term survival under hostile conditions within the host or in response to stress during its environment–animal host infectious cycle (Man, 2011). Fasani and Savageau (2013) have proposed a general model of TA systems in which redundancy of the systems is important for increasing the frequency of persister cells. Evidence is further emerging that TA modules can contribute directly to the virulence repertoire of bacteria (Lobato-Marquez et al., 2016). Conservation of related Fic proteins in isolates of *Arcobacter*, *Bartonella*, *Fusobacterium, Streptococcus, Lachnospiraceae, Prevotella, Gardnerella*, and *Enterococcus* that colonize or cause disease in urogenital or feto-placental tissue in humans and livestock underscores the probable importance of this group of Fic proteins for niche adaptation and pathogenicity. Characterizing the protein-interaction networks of the Fic proteins of *C. fetus* and analogs from other urogenital pathogens will be the next step in understanding these complex multipurpose toxins.

## Author Contributions

HS, SK, GG, and EZ designed the research. CH contributed study resources. HS, SK, BP, LP, BK, PB, DV, and DA performed experiments. HS, SK, BP, FF, and EZ analyzed the data. HS, SK, BP, GG, and EZ wrote the paper. All authors read and approved the final manuscript.

## Conflict of Interest Statement

The authors declare that the research was conducted in the absence of any commercial or financial relationships that could be construed as a potential conflict of interest. The reviewer RO and handling Editor declared their shared affiliation.
