# Supplementary material for: Fic Proteins of Campylobacter fetus subsp. venerealis Form a Network of Functional Toxin–Antitoxin Systems
Source: Front Microbiol. 2017 Oct 17;8:1965. doi: 10.3389/fmicb.2017.01965 (PMC5651007; doi:10.3389/fmicb.2017.01965)
Supplement: Supplementary file 1 [file Table_1.docx]

Supplementary Material

**Fic proteins of *Campylobacter fetus* subsp. *venerealis* form a network of functional toxin-antitoxin systems**

^♯^Hanna Sprenger, ^♯^Sabine Kienesberger, Brigitte Pertschy, Lisa Pöltl, Bettina Konrad, Priya Bhutada, Dina Vorkapic, Denise Atzmüller, Florian Feist, Christoph Högenauer, Gregor Gorkiewicz, and *Ellen L. Zechner

*** Correspondence:** Ellen L. Zechner: ellen.zechner@uni-graz.at

^♯^ **equal contribution to the manuscript**

**Table S1. Bacterial strains and plasmids used in this study.**

| **Strains** | **Description*^a^*** |  | | **References** | |
| --- | --- | --- | --- | --- | --- |
| ***C. fetus* subsp. *venerealis*** |  |  | |  | |
| 84-112 | Bovine isolate, genital secretion, Nal^r^ | | ([Perez-Perez et al., 1986](#_ENREF_6)) | | |
| ***E. coli*** |  |  | |  | |
| DH5α | *endA1 recA1 gyrA96 thi-l hsdR17 supE44 λ- relA1 deoR Δ(lacZYA- argF)- U169 φ80dlacZΔ(M15)* | | | ([Woodcock et al., 1989](#_ENREF_9)) | |
| C41 (DE3) | F – *ompT hsdSB (rB- mB-) gal dcm* (DE3) | | | ([Miroux and Walker, 1996](#_ENREF_5)) | |
| **Plasmids** | **Description*^a^*** | **Primers*** | | | **References** |
| pBAD24 | *E. coli* expression vector, L-arabinose inducible P_BAD_, *bla,* ColE1 origin of replication |  | | | ([Guzman et al., 1995](#_ENREF_2)) |
| pBADKm | *aph* in pBAD24 PvuI site | 15/16 | | | This study |
| pACYC184 | Source of chloramphenicol (*cat*) *gene* |  | | | ([Rose, 1988](#_ENREF_7)) |
| pAR80 | Source of kanamycin (*aph*) gene |  | | | A. Reisner |
| pBAD24-fic1 | *fic1* in pBAD24 MCS KpnI/SalI site | 1/2 | | | This study |
| pBADKm-fic1 | *fic1* in pBADKm MCS KpnI/SalI site | 1/2 | | | This study |
| pBADCm-fic1 | *cat* in pBAD24-fic1 PvuI site | 13/14 | | | This study |
| pBAD24-fic2 | *fic2* in pBAD24 MCS KpnI/SalI site | 3/4 | | | This study |
| pBAD24-fic3 | *fic3* in pBAD24 MCS KpnI/SalI site | 5/6 | | | This study |
| pBAD24-fic4 | *fic4* in pBAD24 MCS KpnI/SalI site | 7/8 | | | This study |
| pBADCm-fic4 | *fic4* in pBADCm-fic1 MCS KpnI/SalI site | 7/8 | | | This study |
| pBADKm-fic4 | *fic4* in pBADKm MCS KpnI/SalI site | 7/8 | | | This study |
| pBAD24-fic4_T209A/E213A | *fic4_T209A/E213A* in pBAD24 MCS KpnI/SalI site | 7/40, 8/41 | | | This study |
| pBADKm-fti3 | *Fti3* in pBADKm MCS KpnI/SalI site | 9/10 | | | This study |
| pBADCm-fti3 | *fti3* in pBADCm MCS KpnI/SalI site | 9/10 | | | This study |
| pBADKm-fti4 | *fti4* in pBADKm MCS KpnI/SalI site | 11/12 | | | This study |
| pBADCm-fic1_S31A/E35A | *fic1_S31A/E35A* in pBADCm-fic1 MCS KpnI/SalI site | 1/17, 2/18 | | | This study |
| pBAD24-fic1_S31A/E35A | *fic1_S31A/E35A* in pBAD24 MCS KpnI/SalI site | 1/17, 2/18 | | | This study |
| pBAD24-fic2_H184A | *fic2_H184A* in pBAD24 MCS KpnI/SalI site | 3/19, 4/20 | | | This study |
| pBAD24-fic2_CffF37 | *fic2* from *Cff* F37 in pBAD24 MCS KpnI/SalI site | 3/4 | | | This study |
| pBAD24-Fic3_H147A | *fic3_H147A* in pBAD24 MCS KpnI/SalI site | 5/22, 6/21 | | |  |
| pFA6a-3xHA | *3xHA* tag |  | | | B. Pertschy |
| pBAD24-fic2-3xHA** | *fic2-3xHA* in pBAD24 MCS KpnI/SalI site | 9/24, 3/25, 3/24 | | | This study |
| pBADKm-fic1-FLAG | *aph* in pBAD24-fic1-FLAG PvuI site | 15/16 | | | This study |
| pBAD24-fic1-FLAG | *fic1-FLAG*  in pBAD24 MCS KpnI/SalI site | 1/38 | | |  |
| pBADKm-fti3-FLAG | *fti3-FLAG* in pBADKm MCS EcoRI/SalI site | 30/31 | | | This study |
| pBAD24-fic3-3xHA | *fic3-3xHA* in pBAD24 MCS NheI/SalI site |  | | | This study |
| pBADKm-fti4-FLAG | *fti4-FLAG* in pBADKm MCS EcoRI/SalI site | 36/37 | | | This study |
| pBAD24-fic4-HA | *fic4-3xHA* in pBAD24 MCS EcoRI/SalI site | 34/35 | | | This study |
| pBAD24-fic4_T209A/E213A-HA | *fic4_T209A/E213A-3xHA* in pBAD24 MCS EcoRI/SalI site | 34/35 | | | This study |
| pBADKm-fic4-FLAG | *fic4-FLAG* in pBADKm MSC EcoRI/SalI site | 34/39 | | | This study |

*Primers used to amplify *fic*-genes and derivatives and resistance markers for insertions (see Table S2);

**Two-step PCR protocol; Nal^r^, nalidixic acid resistance; Km, kanamycin resistance, Cm, chloramphenicol resistance; MCS, multiple cloning site;

**Table S2. Oligonucleotides used in this study.**

| **#** | **Oligonucleotide*^a^*** | **Sequence*^b^* (5´→ 3´)** | **Description and binding site** |
| --- | --- | --- | --- |
| 1 | Fic1_KpnI_f* | TAA***GGTACC***CGATGGCGGTGTAAATTTAGG | *fic1* (nt 4 to 23)*^c^* |
| 2 | Fic1_SalI_r* | CTA***GTCGAC***TTATCTCTCCTTTTCCTTTGAAT | *fic1* (nt 815 to 837)*^c^* |
| 3 | Fic2_KpnI_f* | TAA***GGTACC***CCAAGAACAATATACGGAAATC | *fic2* (nt 4 to 24)*^c^* |
| 4 | Fic2_SalI_r* | CTA***GTCGAC***TTATCTTTCCTTTTCTTTTGATTTT | *fic2* (nt 897 to 921)^c^ |
| 5 | Fic3_KpnI_f* | TAA***GGTACC***CATTGATAAAGTTTTGAAATTTTTAG | ICE_84-112, (nt 14,319 to 14,343)*^d^* |
| 6 | Fic3_SalI_r* | TAA***GTCGAC***TTAACATAAGGATAATCCTAA | ICE_84-112, (nt 14,967 to 14,987)*^d^* |
| 7 | Fic4_KpnI_f* | TAA***GGTACC***CGAATATTTTATAATGTTTCAAGAG | ICE_84-112, (nt 55,420 to 55,443)*^d^* |
| 8 | Fic4_SalI_r* | TAA***GTCGAC***TTATCTGTTATGCTCCAAATT | ICE_84-112, (nt 56,725 to 56,745)*^d^* |
| 9 | Fti3_KpnI_f* | TAA***GGTACC***CACATATCCAGAAGTACATAGTTTAGA | ICE_84-112, (nt 14,090 to 14,115)*^d^* |
| 10 | Fti3_SalI_r* | CTA***GTCGAC***TCAATCATTTATTCCCCATTCTTTTTTATATTCG | ICE_84-112, (nt 14,290 to 14,323)*^d^* |
| 11 | Fti4_KpnI_f | TAA***GGTACC***CAATGTTTATAGCCTAGAAGAAAGTATAGC | ICE_84-112, (nt 55,211 to 55,239)*^d^* |
| 12 | Fti4_SalI_r | TAA***GTCGAC***TTATAAAATATTCCATTGGTGTTTTATTTTTAA | ICE_84-112, (nt 55,400 to 55,432)*^d^* |
| 13 | Cm_PvuI_f | GGATT***CGATCG***GCTAAGGAAGCTAAAATGGAGAAAA | *cat* pACYC184 (nt 210 to 234)*^c^* |
| 14 | Cm_PvuI_r | GGATT***CGATCG***ATTATCACTTATTCAGGCGTAGCAC | *cat* pACYC184 (nt 3743 to 3767)*^c^* |
| 15 | Km_PvuI_f | TAA***CGATCG***GATCAAGAGACAGGATGA GG | *aph* pAR80 |
| 16 | Km_PvuI_r | ATA***CGATCG***TCAGAAGAACTCGTCAAGAAGG | *aph* pAR80 |
| 17 | Fic1_SEAA_r | GTATTGCC**TGC**GATTGCTGT**TGC**ATGATGAGC | *fic1* (nt 82 to 113)*^c^* |
| 18 | Fic1_SEAA_f | GCTCATCAT**GCA**ACAGCAATC**GCA**GGCAATAC | *fic1* (nt 82 to 113)*^c^* |
| 19 | Fic2_H185A_r | TTCTCGAAACGG**TGC**AATTTGCCA | *fic2* (nt 541 to 564)*^c^* |
| 20 | Fic2_H184A_f | TGGCAAATT**GCA**CCGTTTCGAGAA | *fic2* (nt 541 to 564)*^c^* |
| 21 | Fic3_H147A_f | AATGCTCTTGCACCTTTTCGTGAA | *fic3* (nt 473 to 484)*^c^* |
| 22 | Fic3_H147A_r | TTCACGAAAAGGTGCAAGAGCATT | *fic3* (nt 461 to 469)*^c^* |
| 23 | 3xHA_F | ATCGAAGGGCGCTACCCATACGATGTTC | pFA6a-3xHA (nt 61 to 76) |
| 24 | 3xHA_SalI_r | TAA***GTCGAC***TTAATCTGGAACGTCATATGGATAGG | pFA6a-3xHA (nt 122 to 144) |
| 25 | 3xHA_fic2_r | CGTATGGGTAGCGCCCTTCGATTCTTTCCTTTTCTTTTG | *fic2* (nt 902 to 918)*^c^* |
| 26 | cFic2_BamHI_f* | TAA***GGATCC***ATGCAAGAACAATATACGGAAATC | *fic2* (nt 1 to 24)*^c^* |
| 27 | cFic2_PstI_r* | TTT***CTGCAG***CGTTATTTACAAACGCGAATTCC | 23 nt after *fic2^e^* |
| 28 | Fic2_seq_f* | GATGCCTTTCCACAAGGGCTT | *fic2* (nt 316 to 336)*^c^* |
| 29 | Fic2_seq_r* | GGATGAATTTGCCATACCCCG | *fic2* (nt 534 to 554)*^c^* |
| 30 | Fti3_EcoRI_f | GGAG***GAATTC***ACCATGACATATCCAGAAGTACATAG | ICE_84-112, (nt 14,087 to 14,109)*^d^* |
| 31 | Fti3_FLAG_SalI_r | CTA***GTCGAC***TTACTTGTCATCGTCATCCTTGTAATCATCATTTATTCCCCATTCTTTTTTATATTCG | ICE_84-112, (nt 14,290 to 14,320)*^d^* |
| 32 | Fic3_NheI_f | TTAA***GCTAGC***AGGAGGAATTCACCATGATTGATAAAGTTTTG | ICE_84-112, (nt 14,316 to 14,333)*^d^* |
| 33 | Fic3-3xHA_SalI_r | TAA***GTCGAC***TTAAGCGTAATCTGGAACATCGTATGGGTAACATAAGGATAATCCTAATGC | ICE_84-112, (nt 14,964 to 14,986)*^d^* |
| 34 | EcoRI_Fic4_f | TTC***GAATTC***ACCATGGAATATTTTATAATGTTTCAAGA | ICE_84-112, (nt 55,417 to 55,442)*^d^* |
| 35 | SalI_HA_Fic4_r | TAA***GTCGAC***TTAAGCGTAATCTGGAACATCGTATGGGTATCTGTTATGCTCCAAATTTTTATAAT | ICE_84-112, (nt 56,717 to 56,744)*^d^* |

**Table S2 - continued**

| **#** | **Oligonucleotide*^a^*** | **Sequence*^b^* (5´→ 3´)** | **Description and binding site** |
| --- | --- | --- | --- |
| 36 | Fti4_EcoRI_f | GGAG***GAATTC***ACCATGAATGTTTATAGCCTAG | ICE_84-112, (nt 55,208 to 55,226)*^d^* |
| 37 | Fti4_FLAG_SalI_r | TAA***GTCGAC***TTACTTGTCATCGTCATCCTTGTAATCTAAAATATTCCATTGGTGTTTTATTTTTAA | ICE_84-112, (nt 55,400 to 55,429)*^d^* |
| 38 | Fic1_FLAG_r | TAA***GTCGAC***TTACTTATCGTCGTCATCCTTGTAATCTCTCTCCTTTTCCTTTG | *fic1* (nt 818 to 834)*^c^* |
| 39 | Fic4_FLAG_SalI_r | CTA***GTCGAC***TTACTTGTCATCGTCATCCTTGTAATCTCTGTTATGCTCCAAATTTTTATAATTTG | ICE_84-112, (nt 56,714 to 56,742)*^d^* |
| 40 | Fic4_TAEA_f | GGATAAC**GCA**ATGGATAAAG**CAA**ATTCAAAT | ICE_84-112, (nt 56,034 to 56,058)*^d^* |
| 41 | Fic4_TAEA_r | ATTTGAAT**TTG**CTTTATCCAT**TGC**GTTATCC | ICE_84-112, (nt 56,034 to 56,058)*^d^* |

*^a^* Asterisks indicate primers used in the PCR gene screens; *^b^* restriction sites are shown in **bold** and *italics*; codons inducing point mutations are shown in **bold**, and tag overlaps are shown underlined; nt nucleotide; (nt position), in relation to *^c^* nt 1 of the corresponding gene, or *^d^* nt 1 of ICE_84-112; ^e^ primer binds outside of the gene

**Table S3. Templates for Fic protein fold recognition via Phyre2.**

|  | **PDB ID** | **Protein plus source** |
| --- | --- | --- |
| **Fic1** | 3CUC | Protein of unknown function with a Fic domain from *Bacteroides thetaiotaomicron* VPI-5482 |
|  | 3EQX | Fic family protein from *Shewanella oneidensis* MR-1 |
| **Fic2** | 2VZA | cell filamentation protein from *Bartonella henselae* |
|  | 2F6S | cell filamentation protein from *Helicobacter pylori* 26695 |
|  | 2G03 | cell filamentation protein from *Neisseria meningitidis* MC58 |
|  | 3SHG | VbhT from *Bartonella schoenbuchensis* R1 |
|  | 3CUC | protein of unknown function with a Fic domain from *Bacteroides thetaiotaomicron* VPI-5482 |
|  | 3EQX | Fic family protein from *Shewanella oneidensis* MR-1 |
| **Fic3** | 2VZA | cell filamentation protein from *Bartonella henselae* |
|  | 3SHG | VbhT from *Bartonella schoenbuchensis* R1 |
| **Fic4** | 2VZA | cell filamentation protein from *Bartonella henselae* |

**Table S4. Protein Accession Numbers of proteins used in Neighbor joining Tree**

| **Protein** | **Protein Accession Number** |
| --- | --- |
| *Campylobacter* sp RM8964 | WP_086334069.1 |
| Fic *C. coli* 15-537360 | YP_008731335.1 |
| MloA *C. coli* 15-537360 | YP_008747405.1 |
| Fic *C. coli* RM5611 | AHK74499.1 |
| Fic *C. coli* K3 | ETC95351.1 |
| hyp DNA-binding *C. concisus* ATCC51561 | ERJ27647.1 |
| Fic *C. concisus* ATCC 51561 | ERJ27453.1 |
| hyp *C. concisus* UNSW3 | ERJ22768.1 |
| hyp *C. corcagiensis* | WP_025803646.1 |
| Fic *C. cuniculorum* | WP_035175932.1 |
| hyp *C. cuniculorum* | WP_051521686.1 |
| Fic1 *C. fetus* subsp. *venerealis* 84-112 | CDF65254.1 |
| Fic2 *C. fetus* subsp. *venerealis* 84-112 | CDF65253.1 |
| Fic3 *C. fetus* subsp. *venerealis* 84-112 | CDF65920.1 |
| Fic4 *C. fetus* subsp. *venerealis* 84-112 | CDF65967.1 |
| Fic *C. fetus* subsp. *venerealis* 04/554 | AIR78561.1 |
| Fic *C. fetus* subsp. *venerealis* cfvi03/293 | AHE94549.1 |
| Fic *C. fetus* subsp. *venerealis* CCUG33872 | OCS25815.1 |
| Fic *C. fetus* subsp*. venerealis* LMG6570 | OCS28453.1 |
| GlnA *C. fetus* subsp*. venerealis* 84-112 | CDF64852 |
| Doc *C. gracilis* RM3268 | EEV16790.1 |
| Fic *C. helveticus* | WP_082200803.1 |
| Fic *C. jejuni* 32488 | YP_008293623.1 |
| Doc *C. jejuni doylei* 269.97 | YP_001398072.1 |
| MloA *C. jejuni doylei* 269.97 | YP_001398845.1 |
| Fic *C. lari* RM2100 | YP_002576079.1 |
| Doc *C. mucosalis* | KEA46151.1 |
| Fic1 *C. mucosalis* | KEA46573.1 |
| Fic2 *C. mucosalis* | KEA45319.1 |
| hyp *C. mucosalis* | KEA45359.1 |
| Fic *C. rectus* RM3267 | EEF14435.1 |
| Doc *C. upsaliensis* JV21 | EFU71800.1 |
| Fic *C. upsaliensis* JV21 | EFU71140.1 |
| hyp *C. upsaliensis* JV21 | EFU71141.1 |
| Fic *C. ureolyticus* ACS-301-V-Sch3b | EPH07314.1 |
| hyp *C. ureolyticus* ACS-301-V-Sch3b | EPH07928.1 |
| Doc1 *C. ureolyticus* ACS-301-V-Sch3b | EPH10272.1 |
| Doc2 *C. ureolyticus* ACS-301-V-Sch3b | AGJ76579.1 |
| Doc1 *C. ureolyticus* DSM 20703 | AGS56909.1 |
| Doc2 *C. ureolyticus* DSM 20703 | AGS56910.1 |
| Fic *Arcobacter butzleri* ED-1 | YP_005538770.1 |
| VbhT *Bartonella schoenbuchensis* DSM13525 | E6Z0R3.1 |
| BepA *B. henselae* | CAD89506.1 |
| hyp *Enterococcus* sp. Marseille-P2817 | WP_071130534.1 |
| EcFicT *Escherichia coli* | AAA24263.1 |
| Fic *Fusobacterium necrophorum* BFTR-1 | KDE63172.1 |
| Fic *F. nucleatum* | WP_029495389.1 |
| Fic *Gardnerella* *vaginalis* JCP8151B | EPI47133.1 |
| Fic *Helicobacter pylori* P12 | YP_002301757.1 |
| hyp *Lachnospiraceae* bacterium 10-1 | WP_016229701.1 |
| NmFic *Neisseria meningitidis* | 3SN9_A |
| Fic *Prevotella bivia* | WP_04873955.1 |
| Doc *Salmonella. enterica* subsp. *enterica* serovar Thyphimurium | AMM01259.1 |
| Fic *Streptococcus* *agalactiae* STIR-CD-17 | EJZ03953.1 |
| Fic *Yersinia enterocolitica* | CAL11741.1 |

**Table S5. Distribution of *fic* genes in *C. fetus* isolates.**

| **Strain*^a^*** | **Source** | **Reference, country*^b^*** | ***fic1^c^*** | ***fic2^c^*** | ***fic3^c^*** | ***fic4^c^*** | ***fti3^c^*** |
| --- | --- | --- | --- | --- | --- | --- | --- |
| Cff ATCC 27374 (FR) | Brain of sheep fetus, type strain | ATCC, FRA | - | - | - | - | - |
| Cff D (F1) | Bovine | J. Kirpal, GER ([Gorkiewicz et al., 2003](#_ENREF_1)) | - | - | - | - | - |
| Cff B398/2 SK (F5) | Bovine | AUT ([Gorkiewicz et al., 2003](#_ENREF_1)) | - | - | - | - | - |
| Cff B88 (F6) | Bovine | E. Hofer, AUT ([Gorkiewicz et al., 2003](#_ENREF_1)) | - | - | - | - | - |
| Cff H88 (F7) | Bovine | E. Hofer, AUT ([Gorkiewicz et al., 2003](#_ENREF_1)) | - | - | - | - | - |
| Cff S88 (F8) | Bovine | E. Hofer, AUT ([Gorkiewicz et al., 2003](#_ENREF_1)) | - | - | - | - | - |
| Cff 94/4256 (F9) | Aborted bovine fetus | S. Hum, AUS ([Hum et al., 1997](#_ENREF_3)) | - | - | - | - | - |
| Cff 107/4172 (F10) | Aborted bovine placenta | S. Hum, AUS ([Hum et al., 1997](#_ENREF_3)) | - | - | - | - | - |
| Cff 133/4369 (F11) | Aborted bovine fetus | S. Hum, AUS ([Hum et al., 1997](#_ENREF_3)) | - | - | - | - | - |
| Cff 12 (F12) | Human blood | R. Krause, AUT ([Krause et al., 2002](#_ENREF_4)) | - | - | - | - | - |
| Cff CCUG 41395 (F13) | Human | CCUG, SWE | - | - | - | - | - |
| Cff CCUG 43084 (F14) | Human | CCUG, SWE | - | - | - | - | - |
| Cff CCUG 7473 (F15) | Human | CCUG, FRA | - | - | - | - | - |
| Cff CCUG 11286 (F16) | Human blood | CCUG, FRA | - | - | - | - | + |
| Cff CCUG 13315 (F17) | Human blood | CCUG, FIN | - | - | - | - | + |
| Cff CCUG 17694 (F18) | Human blood | CCUG, BEL | - | - | - | - | - |
| Cff CCUG 33671 (F19) | Bull genitals | CCUG, SWE | - | - | - | - | - |
| Cff CCUG 33720 (F20) | Human peritoneal dialysis fluid | CCUG, SWE | - | - | - | - | - |
| Cff CCUG 39963 (F21) | Human blood | CCUG, SWE | - | - | - | - | - |
| Cff CCUG 42302 (F22) | Human blood, diarrhea | CCUG, SWE | - | - | - | - | - |
| Cff CCUG 32676 (F23) | Human | CCUG, CAN | - | - | - | - | - |
| Cff CCUG 30605 (F24) | Human blood and gall | CCUG, SWE | - | - | - | - | - |
| Cff L487 (F25) | Human, diarrhea | G. Feierl, AUT ([Gorkiewicz et al., 2003](#_ENREF_1)) | - | - | - | - | - |
| Cff H97/343 (F27) | Human, diarrhea | G. Feierl, AUT ([Gorkiewicz et al., 2003](#_ENREF_1)) | - | - | - | - | - |
| Cff H97/292 (F28) | Human, diarrhea | G. Feierl, AUT ([Gorkiewicz et al., 2003](#_ENREF_1)) | - | - | - | - | - |
| Cff H00/415 (F29) | Human, diarrhea | G. Feierl, AUT ([Gorkiewicz et al., 2003](#_ENREF_1)) | - | - | - | - | - |
| Cff J 32.844 (F31) | Human, diarrhea | G. Feierl, AUT | - | - | - | - | - |
| Cff J 35.572 (F33) | Human, diarrhea | G. Feierl, AUT | - | - | - | - | - |
| Cff f (3208252420) (F34) | Bull, prepuce | J. Wagenaar, NLD | - | - | - | - | - |
| Cff 5,5,42 (SZ 107) (F35) | Ovine | J. Wagenaar, NLD ([van Bergen et al., 2005](#_ENREF_8)) | - | - | - | - | - |
| Cff 98/v445 (F37) | Bovine | J. Wagenaar, UK ([van Bergen et al., 2005](#_ENREF_8)) | - | + | - | - | - |
| Cff BT36/98 (F38) | Bovine placenta, abortion | J. Wagenaar, UK ([van Bergen et al., 2005](#_ENREF_8)) | + | + | - | - | + |

**Table S5 - continued**

| **Strain*^a^*** | **Source** | **Reference, country*^b^*** | ***fic1^c^*** | ***fic2^c^*** | ***fic3^c^*** | ***fic4^c^*** | ***fti3^c^*** |
| --- | --- | --- | --- | --- | --- | --- | --- |
| Cff IZ-2149-80 (F39) | Bull, prepuce | J. Wagenaar, NLD ([van Bergen et al., 2005](#_ENREF_8)) | - | - | - | - | + |
| Cff 122 (F40) | Ovine | J. Wagenaar, TUR ([van Bergen et al., 2005](#_ENREF_8)) | - | - | - | - | - |
| Cff 89/8/5396 (F41) |  | J. Wagenaar, ZAF | + | - | - | - | - |
| Cff BT10/98 (F42) | Ovine | J. Wagenaar, UK ([van Bergen et al., 2005](#_ENREF_8)) | + | - | - | - | - |
| Cff 3754 (D425) (F44) | Human | USDA, I.Wesley, USA ([van Bergen et al., 2005](#_ENREF_8)) | + | - | - | - | - |
| Cff 82-50 (97-365-1) (F45) |  | USDA, I.Wesley, USA; J. Wagenaar | + | - | - | - | - |
| Cff 84-32 (F47) | Bovine vagina | M. Blaser, USA | - | - | - | - | - |
| Cff 82-40 (F48) | Human blood, renal transplant | M. Blaser, USA | - | - | - | - | - |
| Cfv ATCC 19438 (VR) | Vaginal mucus of heifer, type strain | ATCC, UK | + | + | + | + | + |
| Cfv 1a (V1) | Bovine | E. Pohl, GER ([Gorkiewicz et al., 2003](#_ENREF_1)) | + | + | - | - | + |
| Cfv 3 (V3) | Bovine | J. Kirpal, GER ([Gorkiewicz et al., 2003](#_ENREF_1)) | + | + | - | - | + |
| Cfv G91 (V5) | Bovine | E. Hofer, AUT ([Gorkiewicz et al., 2003](#_ENREF_1)) | + | + | - | - | - |
| Cfv TH15 (V6) | Bovine | E. Hofer, AUT ([Gorkiewicz et al., 2003](#_ENREF_1)) | + | + | - | - | - |
| Cfv 80/4172 (V8) | Bovine | S. Hum, AUS ([Hum et al., 1997](#_ENREF_3)) | + | + | - | - | - |
| Cfv 108/4111 (V9) | Bovine | S. Hum, AUS ([Hum et al., 1997](#_ENREF_3)) | + | + | - | - | + |
| Cfv 121/4401 (V10) | Aborted bovine fetus | S. Hum, AUS ([Hum et al., 1997](#_ENREF_3)) | + | + | - | - | + |
| Cfv CCUG 24260 (V11) | Bovine | CCUG, SWE | + | + | - | - | - |
| Cfv CCUG 33871 (V12) |  | CCUG, CZE | + | + | - | - | + |
| Cfv CCUG 33872 (V13) |  | CCUG, CZE | + | + | - | - | + |
| Cfv CCUG 33901 (V14) |  | CCUG, FRA | + | + | - | - | + |
| Cfv CCUG 33902 (V15) | Bull, prepuce | CCUG, BEL | + | + | - | - | + |
| Cfv CCUG 35146 (V17) | Bovine aborted foetus | CCUG, AUS | + | + | - | - | + |
| Cfv CCUG 33936 (V18) | Cow vagina | CCUG | + | + | - | - | - |
| Cfv CCUG 34394 (V19) | Bovine | CCUG, ARG | + | + | - | - | + |
| Cfv CCUG 33900 (V20) | Cow, abortion product | CCUG | + | + | - | - | + |
| Cfv CCUG 34396 (V21) | Bovine | CCUG, ARG | + | + | - | - | - |
| Cfv NZ 2742-95 (V22) |  | S. Hum, AUS; provided by A. Burnens | + | + | - | - | + |
| Cfv NZ 4264-95 (V23) |  | S. Hum, AUS; provided by A. Burnens | + | + | - | - | + |
| Cfv NZ 4267-95 (V24) | Bovine | S. Hum, AUS; provided by A. Burnens | + | + | - | - | - |
| Cfv NZ 4266-95 (V25) | Bovine | S. Hum, AUS; provided by A. Burnens | + | + | - | - | - |
| Cfv NZ 4268 (V26) | Bovine | S. Hum, AUS; provided by A. Burnens | + | + | - | - | - |
| Cfv NZ 4269-95 (V27) | Bovine | S. Hum, AUS; provided by A. Burnens | + | + | - | - | - |
| Cfv NZ 4270-95 (V28) | Bovine | S. Hum, AUS; provided by A. Burnens | + | + | - | - | - |

| **Table S5 - continued** | | | | | | | | | | | | |  |  |  |
| --- | --- | --- | --- | --- | --- | --- | --- | --- | --- | --- | --- | --- | --- | --- | --- |
| **Strain*^a^*** | | | **Source** | | **Reference, country*^b^*** | | ***fic1^c^*** | | ***fic2^c^*** | ***fic3^c^*** | | ***fic4^c^*** | | ***fti3^c^*** | |
| Cfv NZ 4272-95 (V29) | | Bovine | | S. Hum, AUS; provided by A. Burnens | | + | | | + | | - | | - | | + |
| Cfv NZ 4274-95 (V30) | | Bovine | | S. Hum, AUS; provided by A. Burnens | | + | | | + | | - | | - | | + |
| Cfv CCUG 538 (V31) | | Vaginal mucosa of heifer | | CCUG | | + | | | + | | - | | - | | + |
| Cfv CCUG 11287 (V32) | | Human blood | | CCUG, FRA | | + | | | + | | - | | - | | + |
| Cfv CCUG 7477 (V33) | | Cow abortion product | | CCUG | | + | | | + | | - | | - | | + |
| Cfv CCUG 33899 (V37) | | Vaginal mucosa of heifer | | CCUG | | + | | | + | | - | | - | | + |
| Cfv CCUG 34335 (V42) | |  | | CCUG, URY | | + | | | - | | - | | - | | + |
| Cfv CCUG 34396 (V44) | |  | | CCUG | | + | | | + | | - | | - | | - |
| Cfv CCUG 34395 (V45) | | Bovine | | CCUG, ARG | | + | | | + | | - | | - | | - |
| Cfv 5,5,21 (V46) | | Bovine | | J. Wagenaar, NLD ([van Bergen et al., 2005](#_ENREF_8)) | | + | | | + | | - | | - | | + |
| Cfv 5.5.22 (V47) | | Bovine | | J. Wagenaar, NLD ([van Bergen et al., 2005](#_ENREF_8)) | | + | | | + | | - | | - | | + |
| Cfv 44168 (V49) | | Bovine semen | | J. Wagenaar, NLD ([van Bergen et al., 2005](#_ENREF_8)) | | + | | | + | | - | | - | | + |
| Cfv 97-v549 (V50) | | Bovine | | J. Wagenaar, NLD | | + | | | - | | - | | - | | - |
| Cfv 97-v561 (V51) | | Bovine | | J. Wagenaar, NLD | | + | | | + | | - | | - | | - |
| Cfv 97-v566 (V52) | | Bovine | | J. Wagenaar, NLD | | + | | | - | | - | | - | | - |
| Cfv 97-v571 (V53) | | | Bovine | | J. Wagenaar, NLD | | + | | + | - | | - | | - | |
| Cfv 18156 (V54) | | | Bovine semen | | J. Wagenaar, NLD | | + | | + | - | | - | | + | |
| Cfv v311 (V55) | | | Bovine | | J. Wagenaar, UK ([van Bergen et al., 2005](#_ENREF_8)) | | + | | + | - | | - | | - | |
| Cfv v315 (V56) | | |  | | J. Wagenaar, NLD | | + | | + | - | | - | | - | |
| Cfv LMG 6570 (V57) | | |  | | BCCM/LMG | | + | | + | - | | - | | + | |
| Cfv 87-383 (V58) | | | Bovine | | USDA, L. Tucker, USA; provided by J. Wagenaar | | + | | + | - | | - | | + | |
| Cfv 3287 (ADRI-554) (V59) | | | Bovine | | USDA, I. Wesley (M. Garcia), USA; provided by J. Wagenaar ([van Bergen et al., 2005](#_ENREF_8)) | | + | | + | - | | - | | + | |
| Cfv 3280 (ADRI-502) (V60) | | |  | | USDA, I. Wesley (M. Garcia), USA; provided by J. Wagenaar | | + | | + | + | | + | | + | |
| Cfv 3281(ADRI-510) (V61) | | | Bovine | | USDA, I.Wesley, USA; provided by J. Wagenaar | | + | | + | - | | - | | - | |
| Cfv 3288 (ADRI-555) (V62) | | | Bovine | | USDA, I. Wesley (M. Garcia), USA; provided by J. Wagenaar | | + | | + | + | | - | | + | |
| Cfv 8598 (00-695) (V63) | | | Bovine | | USDA, I.Wesley, USA; provided by J. Wagenaar | | + | | + | - | | - | | + | |
| Cfv 87-71 (V64) | | |  | | J. Wagenaar, NLD | | + | | + | - | | - | | + | |
| Cfv 86-717 (V65) | | |  | | NHLS, L. Tucker, USA; provided by J. Wagenaar | | + | | + | - | | - | | - | |
| Cfv 89-630 (V66) | | |  | | NHLS, L. Tucker, USA; provided by J. Wagenaar | | + | | + | + | | - | | + | |
| Cfv LMG 93.45 (V67) | | | Bovine | | J. Wagenaar, BEL ([van Bergen et al., 2005](#_ENREF_8)) | | + | | + | - | | - | | + | |
| Cfv BT 74/00 (V68) | | | Bovine | | J. Wagenaar, UK ([van Bergen et al., 2005](#_ENREF_8)) | | + | | + | - | | - | | - | |

**Table S5 - continued**

| **Strain*^a^*** | **Source** | **Reference, country*^b^*** | ***fic1^c^*** | ***fic2^c^*** | ***fic3^c^*** | ***fic4^c^*** | ***fti3^c^*** |
| --- | --- | --- | --- | --- | --- | --- | --- |
| Cfv 110800-21-2 (V75) | Bull, prepuce | J. Wagenaar, NLD | + | + | - | - | - |
| Cfv 040900-24a (V76) | Bull, prepuce | J. Wagenaar, NLD | + | + | - | - | - |
| Cfv 110900-17a (V77) | Bull, prepuce | J. Wagenaar, NLD | + | + | - | - | - |
| Cfv 511 (V78) | Bovine semen | J. Wagenaar, HUN | + | + | + | - | + |
| Cfv 515 (V79) | Bovine semen | J. Wagenaar, NLD | + | + | + | - | + |
| Cfv 84-112 (V81) | Bovine | M. Blaser, USA (Perez-Perez et al., 1986[Perez-Perez et al., 1986](#_ENREF_6)) | + | + | + | + | + |

*^a^* Abbreviations: Cff, *Campylobacter fetus* subsp. *fetus*; Cfv, *Campylobacter fetus* subsp. *venerealis*; our strain collection designation in parentheses

*^b^* Dr. E. Pohl, Deutsche Veterinärmedizinische Gesellschaft, Aulendorf, Germany; Dr. J. Kirpal, Inst. für Mikrobiologie und Tierseuchen der Tierärztlichen Hochschule Hannover, Germany; Dr. E. Hofer, Bundesanstalt für Tierseuchenbekämpfung, Wien, Austria; Dr. R. Krause, Klinische Abteilung f. Pulmonologie & Infektiologie, Universitätsklinik für Innere Medizin, Medizinische Universität, Graz, Austria; Dr. G. Feierl, Institut für Hygiene, Medizinische Universität, Graz, Austria; Dr. J. Wagenaar, Department of Infectious Diseases & Immunology, Utrecht University, The Netherlands; Dr. S. Hum, Camden, Australia; Dr. M. J. Blaser, Department of Medicine & Microbiology, New York School of Medicine, USA; Dr. I. Wesley, USDA, Iowa, USA; Dr. A. Burnens, MCL Laboratories, Dudingen, Switzerland.

Abbreviations: ATCC, American Type Collection; CCUG, Culture Collection, University of Göteborg, Sweden; BCCM/LMG; Bacteria Collection, Laboratorium voor Microbiologie, Universiteit Gent, Belgium; NHLS, National Health Laboratory Service; GER, Germany, AUT, Austria, AUS, Australia; SWE, Sweden; FRA, France; FIN, Finland; BEL, Belgium; CAN, Canada; NLD, The Netherlands; UK, United Kingdom; TUR, Turkey; ZAF, South Africa; CZE, Czech Republic; ARG, Argentina; URY, Uruguay; HUN, Hungary.

*^c^* blue fields, antitoxin *fic1* but no *fic2* present; green fields, antitoxin *fti3* but no *fic3* present; red, *fic2* but no antitoxin *fic1* present

**REFERENCES**

Gorkiewicz, G., Feierl, G., Schober, C., Dieber, F., Kofer, J., Zechner, R., et al. (2003). Species-specific identification of campylobacters by partial 16S rRNA gene sequencing. *J Clin Microbiol* 41(6)**,** 2537-2546.

Guzman, L.M., Belin, D., Carson, M.J., and Beckwith, J. (1995). Tight regulation, modulation, and high-level expression by vectors containing the arabinose PBAD promoter. *J Bacteriol* 177(14)**,** 4121-4130.

Hum, S., Quinn, K., Brunner, J., and On, S.L. (1997). Evaluation of a PCR assay for identification and differentiation of *Campylobacter fetus* subspecies. *Aust Vet J* 75(11)**,** 827-831.

Krause, R., Ramschak-Schwarzer, S., Gorkiewicz, G., Schnedl, W.J., Feierl, G., Wenisch, C., et al. (2002). Recurrent septicemia due to *Campylobacter* *fetus* and *Campylobacter lari* in an immunocompetent patient. *Infection* 30(3)**,** 171-174.

Miroux, B., and Walker, J.E. (1996). Over-production of proteins in *Escherichia coli*: mutant hosts that allow synthesis of some membrane proteins and globular proteins at high levels. *J Mol Biol* 260(3)**,** 289-298. doi: S0022-2836(96)90399-X [pii]10.1006/jmbi.1996.0399.

Perez-Perez, G.I., Blaser, M.J., and Bryner, J.H. (1986). Lipopolysaccharide structures of *Campylobacter fetus* are related to heat-stable serogroups. *Infect Immun* 51(1)**,** 209-212.

Rose, R.E. (1988). The nucleotide sequence of pACYC184. *Nucleic Acids Res* 16(1)**,** 355.

van Bergen, M.A., Dingle, K.E., Maiden, M.C., Newell, D.G., van der Graaf-Van Bloois, L., van Putten, J.P., et al. (2005). Clonal nature of *Campylobacter fetus* as defined by multilocus sequence typing. *J Clin Microbiol* 43(12)**,** 5888-5898. doi: 10.1128/JCM.43.12.5888-5898.2005.

Woodcock, D.M., Crowther, P.J., Doherty, J., Jefferson, S., DeCruz, E., Noyer-Weidner, M., et al. (1989). Quantitative evaluation of *Escherichia coli* host strains for tolerance to cytosine methylation in plasmid and phage recombinants. *Nucleic Acids Res* 17(9)**,** 3469-3478.
